# Two new *Erythrophylloporus* species (Boletaceae) from Thailand, with two new combinations of American species

**DOI:** 10.3897/mycokeys.55.34570

**Published:** 2019-06-21

**Authors:** Santhiti Vadthanarat, Mario Amalfi, Roy E. Halling, Victor Bandala, Saisamorn Lumyong, Olivier Raspé

**Affiliations:** 1 Department of Biology, Faculty of Science, Chiang Mai University, Chiang Mai, 50200, Thailand; 2 PhD’s Degree Program in Biodiversity and Ethnobiology, Department of Biology, Faculty of Science, Chiang Mai University, Chiang Mai, 50200, Thailand; 3 Academy of Science, The Royal Society of Thailand, Bangkok 10300, Thailand; 4 Botanic Garden Meise, Nieuwelaan 38, 1860 Meise, Belgium; 5 New York Botanical Garden, 2900 Southern Blvd, Bronx, New York 10458, USA; 6 Red Biodiversidad y Sistemática, Instituto de Ecología A.C., P.O. Box 63, Xalapa, Veracruz, 91000, México; 7 Fédération Wallonie–Bruxelles, Service général de l’Enseignement universitaire et de la Recherche scientifique, Rue A. Lavallée 1, 1080 Bruxelles, Belgium; 8 Center of Excellence in Microbial Diversity and Sustainable Utilization, Faculty of Science, Chiang Mai University, Chiang Mai, 50200, Thailand; 9 Academy of Science, The Royal Society of Thailand, Bangkok, 10300, Thailand

**Keywords:** *atp*6, *cox*3, Taxonomy, *
Phylloporus
*, *Pulveroboletus* group, multigene phylogeny, Boletales, Southeast Asia

## Abstract

*Erythrophylloporus* is a lamellate genus in the family Boletaceae that has been recently described from China based on *E.cinnabarinus*, the only known species. Typical characters of *Erythrophylloporus* are reddish-orange to yellowish-red basidiomata, including lamellae, bright yellow basal mycelium and smooth, broadly ellipsoid, ellipsoid to nearly ovoid basidiospores. During our survey on diversity of Boletaceae in Thailand, several yellowish-orange to reddish- or brownish-orange lamellate boletes were collected. Based on both morphological evidence and molecular analyses of a four-gene dataset (*atp*6, *tef*1, *rpb*2 and *cox*3), they were recognised as belonging in *Erythrophylloporus* and different from the already known species. Two new species, *E.paucicarpus* and *E.suthepensis* are therefore introduced from Thailand with detailed descriptions and illustrations. Moreover, two previously described *Phylloporus* species, *P.aurantiacus* and *P.fagicola*, were also revised and recombined in *Erythrophylloporus*. A key to all known *Erythrophylloporus* species is provided.

## Introduction

Most fungi in the family Boletaceae are pileate-stipitate with poroid hymenophore, but some have a lamellate hymenophore. Lamellate Boletaceae are currently classified in four genera, *Phylloporus* Quél, which contains about 84 species worldwide, *Phylloboletellus* Singer from South America and Mexico, the two recently described genera *Phylloporopsis* Angelini et al., from the New World and *Erythrophylloporus* Ming Zhang & T.H. Li from Asia, each of which circumscribes only one species (http://www.indexfungorum.org, Farid et al. 2018; Zhang and Li 2018).

The genus *Erythrophylloporus* was recently described from China, with *E.cinnabarinus* Ming Zhang & T.H. Li as the type species. According to Zhang & Li (2018), the typical characters of the genus are orange to reddish-orange basidiomes, reddish-orange to yellowish-red lamellae turning greyish-green when bruised, bright yellow to orange yellow context staining blackish-blue to dark blue when exposed, bright yellow basal mycelium, smooth and broadly ellipsoid to nearly ovoid basidiospores and yellowish-brown pigmented cystidia. During our survey on the diversity of Boletaceae in Thailand, several collections of lamellate boletes were discovered. Some collections were recognised to belong to *Erythrophylloporus* by possessing yellowish-orange to deep orange to reddish-orange basidiomata with bright yellow basal mycelium and smooth basidiospores. We also found that two described *Phylloporus* species, *P.aurantiacus* Halling & G.M. Mueller from Costa Rica and *P.fagicola* Montoya & Bandala from Mexico (Halling et al. 1999, Montoya and Bandala 2011), share similar morphological characters with the genus *Erythrophylloporus*, but until now, have not been included in a molecular phylogeny. In this study, a combination of phylogenetic and morphological evidence indicated that our Thai collections were new species, that, together with the two aforementioned American *Phylloporus* species, belong in *Erythrophylloporus*. Therefore, we introduce two new species with detailed descriptions and illustrations and propose two new combinations. As some of the species we studied have some characters that do not fit with the protologue of the genus, we emend its description.

## Materials and methods

### Specimen collecting

Specimens were obtained and photographed from community forests and Doi Suthep-Pui National Park, Chiang Mai Province, northern Thailand during the rainy season in 2015 to 2016. The specimens were wrapped in aluminium foil and taken to the laboratory. After description of macroscopic characters, all specimens were dried in an electric drier at 45–50 °C. Examined specimens were deposited in the herbaria CMUB, MFLU, BKF or BR (Index Herbariorum; Thiers, continuously updated).

### Morphological studies

Macroscopic descriptions were made based on detailed field notes and photos of fresh basidiomata. Colour codes follow Kornerup and Wanscher (1978). Macrochemical reactions (colour reactions) of fresh basidiomata were determined using 10% potassium hydroxide (KOH) and 28–30% ammonium hydroxide (NH_4_OH) in water. Microscopic structures were observed from dried specimens mounted in 5% KOH, NH_4_OH, Melzer’s reagent or 1% ammoniacal Congo red. A minimum of 50 basidiospores, 20 basidia and 20 cystidia were randomly measured at 1000× with a calibrated ocular micrometer using an Olympus CX51 microscope. The notation ‘[*m*/*n*/*p*]’ represents the number of basidiospores *m* measured from *n* basidiomata of *p* collections. Dimensions of microscopic structures are presented in the following format: (*a*–)*b*–*c*–*d* (–*e*), in which *c* represents the average, *b* the 5^th^ percentile, *d* the 95^th^ percentile and *a* and *e* the minimum and maximum values, respectively. *Q*, the length/width ratio, is presented in the same format. A section of the pileus surface was radially and perpendicularly cut at a point halfway between the centre and margin of the pileus. Sections of stipitipellis were taken from halfway up the stipe and longitudinally cut, perpendicularly to the surface. All microscopic features were drawn by free hand using an Olympus Camera Lucida model U−DA, fitted to the microscope cited above. For scanning electron microscopy (SEM), a spore print was mounted on to a SEM stub with double-sided tape. The sample was coated with gold, examined and photographed with a JEOL JSM–5910 LV SEM (JEOL, Japan).

### DNA isolation, PCR amplification and DNA sequencing

Genomic DNA was extracted from fresh tissue preserved in CTAB or about 10–15 mg of dried specimens using a CTAB isolation procedure adapted from Doyle and Doyle (1990). Portions of the genes *atp*6, *tef*1, *rpb*2 and *cox*3 were amplified by the polymerase chain reaction (PCR) technique. The tailed primers ATP6-1M40F and ATP6-2M (Raspé et al. 2016) and the primer pairs EF1-983F/EF1-2218R (Rehner and Buckley 2005) and bRPB2-6F/bRPB2-7.1R (Matheny 2005) were used to amplify *atp*6, *tef*1 and *rpb*2, respectively. PCR conditions were the same as in Raspé et al. (2016). Part of the mitochondrial gene *cox*3 was amplified with the primers COX3M1-F and COX3M1-R (Vadthanarat et al. 2019), using KAPA2G™ Robust HotStart polymerase (Kapa Biosystems, Wilmington, MA, USA) and the following PCR programme: 2 min 30 s at 95 °C; 35 cycles of 25 s at 95 °C, 30 s at 48 °C, 30 s at 72 °C; 3 min at 72 °C. PCR products were purified by adding 1 U of Exonuclease I and 0.5 U FastAP Alkaline Phosphatase (Thermo Scientific, St. Leon-Rot, Germany) and incubated at 37 °C for 1 h, followed by inactivation at 80 °C for 15 min. Sequencing was performed by Macrogen Inc. (Korea and The Netherlands) with PCR primers, except for *atp*6, for which universal primers M13F-pUC(-40) and M13F(-20) were used; for *tef*1, additional sequencing was performed with two internal primers, EF1-1577F and EF1-1567R (Rehner and Buckley 2005).

### Alignment and phylogeny inference

The sequences were assembled in GENEIOUS Pro v. 6.0.6 (Biomatters) and introns were removed prior to alignment, based on the amino acid sequence of previously published sequences. All sequences, including sequences from GenBank, were aligned using MAFFT version 7 (Katoh and Standley 2013) on the server accessed at http://mafft.cbrE.jp/alignment/server/.

Maximum Likelihood (ML) phylogenetic tree inference was performed using RAxML-HPC2 version 8.2.10 (Stamatakis 2006) on the CIPRES web portal (Miller et al. 2009). The phylogenetic tree was inferred from a four-partitions combined dataset, using the GTRCAT model with 25 categories. Two *Buchwaldoboletus* and nine *Chalciporus* species from subfamily Chalciporoideae were used as the outgroup. Statistical support of clades was obtained with 1,000 rapid bootstrap replicates.

For Bayesian Inference (BI), the best-fit model of substitution amongst those implementable in MrBayes was estimated separately for each gene using jModeltest (Darriba et al. 2012) on the CIPRES portal, based on the Bayesian Information Criterion (BIC). The selected models were GTR+I+G for *atp*6 and *cox*3, SYM+I+G for *tef*1 and K80+I+G for *rpb*2. Partitioned Bayesian analysis was performed with MrBayes 3.2 (Ronquist et al. 2012) on the CIPRES portal. Two runs of five chains were run for 15,000,000 generations and sampled every 1,000 generations. The chain temperature was decreased to 0.02 to improve convergence. At the end of the run, the average deviation of split frequencies was 0.007058 and the Potential Scale Reduction Factor (PSRF) values of all parameters were close to 1. The burn-in phase (25%) was estimated by checking the stationarity in the plot generated by the sump command.

## Results

### Phylogenetic analysis

**Figure 1.**
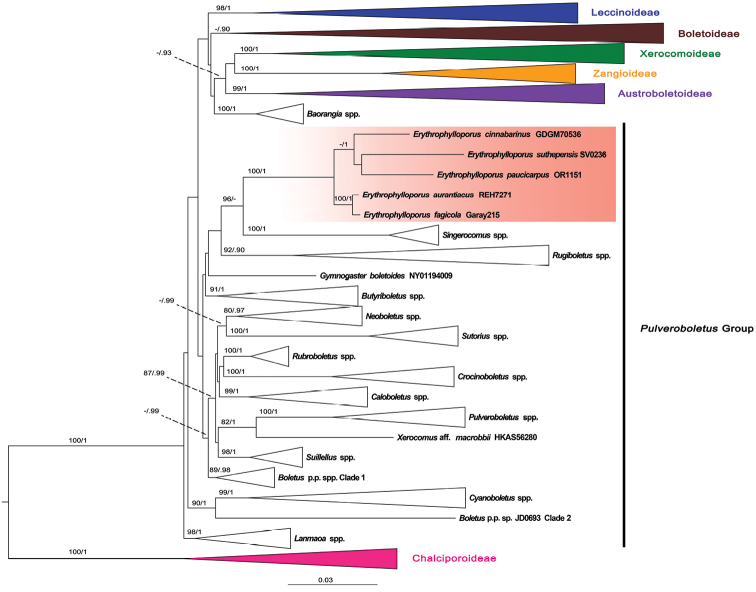
Phylogenetic tree inferred from the four-gene dataset (*atp*6, *rpb*2, *tef*1 and *cox*3), including *Erythrophylloporus* species and selected Boletaceae using Maximum Likelihood and Bayesian Inference methods (ML tree is presented). The two *Buchwaldoboletus* and nine *Chalciporus* species in subfamily Chalciporoideae were used as outgroup. Most of the taxa not belonging to the *Pulveroboletus* group were collapsed into subfamilies. All generic clades, including one undescribed generic clade in *Pulveroboletus* group that were highly supported, were also collapsed. Bootstrap support values (BS ≥ 70%) and posterior probabilities (PP ≥ 0.90) are shown above the supported branches.

Twenty-five sequences were newly generated and deposited in GenBank (Table 1). The sequences from three specimens, OR0689, OR1135 (*E.paucicarpus*) and OR0615B (*E.suthepensis*), were not included in our phylogenic analyses because they were identical to the sequences of the type specimens of *E.paucicarpus* and *E.suthepensis*. The alignment contained 906 sequences (179 for *atp*6, 313 for *tef*1, 279 for *rpb*2, 135 for *cox*3) from 315 voucher specimens and was 2946 characters long (TreeBase number 24078). ML and BI trees showed similar topologies without any supported conflict (Bootstrap Support values, BS ≥ 70% and posterior probabilities, PP ≥ 0.90; Fig. 1). The four-gene phylogram indicated that the included taxa formed seven major clades, representing the Austroboletoideae, Boletoideae, Chalciporoideae, Leccinoideae, Xerocomoideae, Zangioideae and the *Pulveroboletus* group. *Erythrophylloporuscinnabarinus* (typus generis) grouped with the two new *Erythrophylloporus* species, *E.paucicarpus* and *E.suthepensis*, in a highly supported clade (BS = 100% and PP = 1). The two New World *Phylloporus* species (*P.aurantiacus* voucher REH7271 and *P.fagicola* voucher Garay215) also clustered in the *Erythrophylloporus* clade indicating that they are close relatives. *Erythrophylloporus* formed a clade sister to the genus *Singerocomus* T.W. Henkel & M.E. Sm. with high Bootstrap support (96%) but low posterior probability support (0.86) within the *Pulveroboletus* group. Some undescribed species formed two different generic clades in the *Pulveroboletus* group. *Boletus* p.p. spp. clade 1 contains two specimens, HKAS63598 and HKAS9660, named “*Boletus* sp.” in Wu et al. (2016), as well as two of our specimens, OR0832 and OR1002. *Boletus* p.p. sp. clade 2 contains a single African specimen, JD0693, sister to and morphologically different from *Cyanoboletus*.

**Table 1.** List of collections used in this study, with origin and GenBank accession numbers. Newly generated sequences are presented in bold.

Species	Voucher	Origin	*atp*6	*tef*1	*rpb*2	*cox*3	References
Afroboletus aff. multijugus	JD671	Burundi	MH614651	MH614700	MH614747	MH614794	Vadthanarat et al. 2019
* Afroboletus costatisporus *	ADK4644	Togo	KT823958	KT824024	KT823991	MH614795*	Raspé et al. 2016; *Vadthanarat et al. 2019
* Afroboletus luteolus *	ADK4844	Togo	MH614652	MH614701	MH614748	MH614796	Vadthanarat et al. 2019
* Aureoboletus catenarius *	HKAS54467	China	–	KT990711	KT990349	–	Wu et al. 2016
* Aureoboletus duplicatoporus *	HKAS50498	China	–	KF112230	KF112754	–	Wu et al. 2014
* Aureoboletus gentilis *	ADK4865	Belgium	KT823961	KT824027	KT823994	MH614797*	Raspé et al. 2016; *Vadthanarat et al. 2019
* Aureoboletus mirabilis *	HKAS57776	China	–	KF112229	KF112743	–	Wu et al. 2014
* Aureoboletus moravicus *	VDKO1120	Belgium	MG212528	MG212573	MG212615	MH614798*	Vadthanarat et al. 2018; *Vadthanarat et al. 2019
* Aureoboletus nephrosporus *	HKAS67931	China	–	KT990720	KT990357	–	Wu et al. 2016
* Aureoboletus projectellus *	AFTOL-ID-713	USA	DQ534604*	AY879116	AY787218	–	*Binder & Hibbett 2006; Binder, Matheny & Hibbett, Unpublished
* Aureoboletus shichianus *	HKAS76852	China	–	KF112237	KF112756	–	Wu et al. 2014
*Aureoboletus* sp.	HKAS56317	China	–	KF112239	KF112753	–	Wu et al. 2014
*Aureoboletus* sp.	OR0245	China	MH614653	MH614702	MH614749	MH614799	Vadthanarat et al. 2019
*Aureoboletus* sp.	OR0369	Thailand	MH614654	MH614703	MH614750	MH614800	Vadthanarat et al. 2019
* Aureoboletus thibetanus *	HKAS76655	China	–	KF112236	KF112752	–	Wu et al. 2014
* Aureoboletus thibetanus *	AFTOL-ID-450	China	DQ534600*	DQ029199	DQ366279	–	*Binder and Hibbett 2006; Unpublished
* Aureoboletus tomentosus *	HKAS80485	China	–	KT990715	KT990353	–	Wu et al. 2016
* Aureoboletus viscosus *	OR0361	Thailand	MH614655	MH614704	MH614751	MH614801	Vadthanarat et al. 2019
* Aureoboletus zangii *	HKAS74766	China	–	KT990726	KT990363	–	Wu et al. 2016
Austroboletus cf. dictyotus	OR0045	Thailand	KT823966	KT824032	KT823999	MH614802*	Raspé et al. 2016; *Vadthanarat et al. 2019
Austroboletus cf. subvirens	OR0573	Thailand	MH614656	MH614705	MH614752	MH614803	*Vadthanarat et al. 2019
* Austroboletus eburneus *	REH9487	Australia	–	JX889708	–	–	Halling et al. 2012b
* Austroboletus olivaceoglutinosus *	HKAS57756	China	–	KF112212	KF112764	–	Wu et al. 2014
*Austroboletus* sp.	HKAS59624	China	–	KF112217	KF112765	–	Wu et al. 2014
*Austroboletus* sp.	OR0891	Thailand	MH614657	MH614706	MH614753	MH614804	Vadthanarat et al. 2019
* Baorangia pseudocalopus *	HKAS63607	China	–	KF112167	KF112677	–	Wu et al. 2014
* Baorangia pseudocalopus *	HKAS75739	China	–	KJ184570	KM605179	–	Wu et al. 2015
* Baorangia pseudocalopus *	HKAS75081	China	–	KF112168	KF112678	–	Wu et al. 2014
* Baorangia rufomaculata *	BOTH4144	USA	MG897415	MG897425	MG897435	MH614805*	Phookamsak et al. 2019; *Vadthanarat et al. 2019
* Baorangia major *	OR0209	Thailand	MG897421	MG897431	MG897441	MK372295*	Phookamsak et al. 2019; *Vadthanarat et al. 2019
Boletellus aff. ananas	NY815459	Costa Rica	–	KF112308	KF112760	–	Wu et al. 2014
Boletellus aff. emodensis	OR0061	Thailand	KT823970	KT824036	KT824003	MH614806*	Raspé et al. 2016; *Vadthanarat et al. 2019
* Boletellus ananas *	K(M)123769	Belize	MH614658	MH614707	MH614754	MH614807	Vadthanarat et al. 2019
*Boletellus* sp.	OR0621	Thailand	MG212529	MG212574	MG212616	MH614808*	Vadthanarat et al. 2018; *Vadthanarat et al. 2019
*Boletellus* sp.	HKAS58713	China	–	KF112307	KF112759	–	Wu et al. 2014
*Boletellus* sp.	HKAS59536	China	–	KF112306	KF112758	–	Wu et al. 2014
* Boletus aereus *	VDKO1055	Belgium	MG212530	MG212575	MG212617	MH614809*	Vadthanarat et al. 2018; *Vadthanarat et al. 2019
* Boletus albobrunnescens *	OR0131	Thailand	KT823973	KT824039	KT824006	MH614810*	Raspé et al. 2016; *Vadthanarat et al. 2019
* Boletus botryoides *	HKAS53403	China	–	KT990738	KT990375	–	Wu et al. 2016
* Boletus edulis *	HMJAU4637	Russia	–	KF112202	KF112704	–	Wu et al. 2014
* Boletus edulis *	VDKO0869	Belgium	MG212531	MG212576	MG212618	MH614811*	Vadthanarat et al. 2018; *Vadthanarat et al. 2019
*Boletus* p.p. sp	JD0693	Burundi	MH645583	MH645591	MH645599	–	Vadthanarat et al. 2019
*Boletus* p.p. sp.	OR0832	Thailand	MH645584	MH645592	MH645600	MH645605	Vadthanarat et al. 2019
*Boletus* p.p. sp.	OR1002	Thailand	MH645585	MH645593	MH645601	MH645606	Vadthanarat et al. 2019
* Boletus pallidus *	BOTH4356	USA	MH614659	MH614708	–	MH614812	Vadthanarat et al. 2019
* Boletus pallidus *	TDB-1231-Bruns	–	AF002142	–	–	AF002154	Kretzer and Bruns 1999
* Boletus reticuloceps *	HKAS57671	China	–	KF112201	KF112703	–	Wu et al. 2014
*Boletus* s.s. sp.	OR0446	China	MG212532	MG212577	MG212619	MH614813*	Vadthanarat et al. 2018; *Vadthanarat et al. 2019
*Boletus* sp.	HKAS59660	China	–	KF112153	KF112664	–	Wu et al. 2014
*Boletus* sp.	HKAS63598	China	–	KF112152	KF112663	–	Wu et al. 2014
* Boletus violaceofuscus *	HKAS62900	China	–	KF112219	KF112762	–	Wu et al. 2014
* Borofutus dhakanus *	HKAS73789	Bangladesh	–	JQ928576	JQ928597	–	Hosen et al. 2013
* Borofutus dhakanus *	OR0345	Thailand	MH614660	MH614709	MH614755	MH614814	Vadthanarat et al. 2019
* Buchwaldoboletus lignicola *	HKAS76674	China	–	KF112277	KF112819	–	Wu et al. 2014
* Buchwaldoboletus lignicola *	VDKO1140	Belgium	MH614661	MH614710	MH614756	MH614815	Vadthanarat et al. 2019
* Butyriboletus appendiculatus *	VDKO0193b	Belgium	MG212537	MG212582	MG212624	MH614816*	Vadthanarat et al. 2018; *Vadthanarat et al. 2019
Butyriboletus cf. roseoflavus	OR0230	China	KT823974	KT824040	KT824007	MH614819*	Raspé et al. 2016; *Vadthanarat et al. 2019
* Butyriboletus frostii *	NY815462	USA	–	KF112164	KF112675	–	Wu et al. 2014
* Butyriboletus pseudoregius *	VDKO0925	Belgium	MG212538	MG212583	MG212625	MH614817*	Vadthanarat et al. 2018; *Vadthanarat et al. 2019
* Butyriboletus pseudospeciosus *	HKAS63513	China	–	KT990743	KT990380	–	Wu et al. 2016
* Butyriboletus roseoflavus *	HKAS54099	China	–	KF739779	KF739703	–	Wu et al. 2014
* Butyriboletus roseopurpureus *	BOTH4497	USA	MG897418	MG897428	MG897438	MH614818*	Phookamsak et al. 2019; *Vadthanarat et al. 2019
*Butyriboletus* sp.	HKAS52525	China	–	KF112163	KF112671	–	Wu et al. 2014
*Butyriboletus* sp.	HKAS59814	China	–	KF112199	KF112699	–	Wu et al. 2014
*Butyriboletus* sp.	HKAS57774	China	–	KF112155	KF112670	–	Wu et al. 2014
* Butyriboletus subsplendidus *	HKAS50444	China	–	KT990742	KT990379	–	Wu et al. 2016
* Butyriboletus yicibus *	HKAS55413	China	–	KF112157	KF112674	–	Wu et al. 2014
* Caloboletus calopus *	ADK4087	Belgium	MG212539	KJ184566	KP055030	MH614820	Vadthanarat et al. 2018; Zhao et al. 2014a; Zhao et al. 2014b; Vadthanarat et al. 2019
* Caloboletus inedulis *	BOTH3963	USA	MG897414	MG897424	MG897434	MH614821*	Phookamsak et al. 2019; *Vadthanarat et al. 2019
* Caloboletus panniformis *	HKAS55444	China	–	KF112165	KF112666	–	Wu et al. 2014
* Caloboletus radicans *	VDKO1187	Belgium	MG212540	MG212584	MG212626	MH614822*	Vadthanarat et al. 2018; *Vadthanarat et al. 2019
*Caloboletus* sp.	HKAS53353	China	–	KF112188	KF112668	–	Wu et al. 2014
*Caloboletus* sp.	OR0068	Thailand	MH614662	MH614711	MH614757	MH614823	Vadthanarat et al. 2019
* Caloboletus yunnanensis *	HKAS69214	China	–	KJ184568	KT990396	–	Zhao et al. 2014a; Wu et al. 2016
Chalciporus aff. piperatus	OR0586	Thailand	KT823976	KT824042	KT824009	MH614824*	Raspé et al. 2016; *Vadthanarat et al. 2019
Chalciporus aff. rubinus	OR0139	China	MH614663	MH614712	MH614758	–	Vadthanarat et al. 2019
* Chalciporus africanus *	JD517	Cameroon	KT823963	KT824029	KT823996	MH614825*	Raspé et al. 2016; *Vadthanarat et al. 2019
* Chalciporus piperatus *	VDKO1063	Belgium	MH614664	MH614713	MH614759	MH614826	Vadthanarat et al. 2019
* Chalciporus rubinus *	AF2835	Belgium	KT823962	KT824028	KT823995	–	Raspé et al. 2016
*Chalciporus* sp.	HKAS53400	China	–	KF112279	KF112821	–	Wu et al. 2014
*Chalciporus* sp.	HKAS74779	China	–	KF112278	KF112820	–	Wu et al. 2014
*Chalciporus* sp.	OR0363	Thailand	MH645586	MH645594	MH645602	MH645607	Vadthanarat et al. 2019
*Chalciporus* sp.	OR0373	Thailand	MH645587	MH645595	MH645603	MH645608	Vadthanarat et al. 2019
*Chiua* sp.	OR0141	China	MH614665	MH614714	MH614760	MH614827	Vadthanarat et al. 2019
* Chiua virens *	HKAS76678	China	–	KF112272	KF112793	–	Wu et al. 2014
* Chiua virens *	OR0266	China	MG212541	MG212585	MG212627	MH614828*	Vadthanarat et al. 2018; *Vadthanarat et al. 2019
* Chiua viridula *	HKAS74928	China	–	KF112273	KF112794	–	Wu et al. 2014
Crocinoboletus cf. laetissimus	OR0576	Thailand	KT823975	KT824041	KT824008	MH614833*	Raspé et al. 2016; *Vadthanarat et al. 2019
* Crocinoboletus rufoaureus *	HKAS53424	China	–	KF112206	KF112710	–	Wu et al. 2014
* Cyanoboletus brunneoruber *	OR0233	China	MG212542	MG212586	MG212628	MH614834*	Vadthanarat et al. 2018; *Vadthanarat et al. 2019
* Cyanoboletus instabilis *	HKAS59554	China	–	KF112186	KF112698	–	Wu et al. 2014
* Cyanoboletus pulverulentus *	RW109	Belgium	KT823980	KT824046	KT824013	MH614835*	Raspé et al. 2016; *Vadthanarat et al. 2019
* Cyanoboletus sinopulverulentus *	HKAS59609	China	–	KF112193	KF112700	–	Wu et al. 2014
*Cyanoboletus* sp.	OR0257	China	MG212543	MG212587	MG212629	MH614836*	Vadthanarat et al. 2018; *Vadthanarat et al. 2019
*Cyanoboletus* sp.	HKAS76850	China	–	KF112187	KF112697	–	Wu et al. 2014
*Cyanoboletus* sp.	OR0322	Thailand	MH614673	MH614722	MH614768	MH614837	Vadthanarat et al. 2019
*Cyanoboletus* sp.	OR0491	China	MH614674	MH614723	MH614769	MH614838	Vadthanarat et al. 2019
*Cyanoboletus* sp.	OR0961	Thailand	MH614675	MH614724	MH614770	MH614839	Vadthanarat et al. 2019
* Erythrophylloporus aurantiacus *	REH7271	Costa Rica	**MH614666**	**MH614715**	**MH614761**	**MH614829**	This study
* Erythrophylloporus cinnabarinus *	GDGM70536	China	–	MH378802	MH374035	–	Zhang and Li 2018
* Erythrophylloporus fagicola *	Garay215	Mexico	**MH614667**	**MH614716**	**MH614762**	**MH614830**	This study
* Erythrophylloporus paucicarpus *	OR1151	Thailand	**MH614670**	**MH614719**	**MH614765**	**MH614831**	This study
* Erythrophylloporus paucicarpus *	OR0689	Thailand	**MH614668**	**MH614717**	**MH614763**	–	This study
* Erythrophylloporus paucicarpus *	OR1135	Thailand	**MH614669**	**MH614718**	**MH614764**	–	This study
* Erythrophylloporus suthepensis *	SV0236	Thailand	**MH614672**	**MH614721**	**MH614767**	**MH614832**	This study
* Erythrophylloporus suthepensis *	OR0615B	Thailand	**MH614671**	**MH614720**	**MH614766**	–	This study
* Fistulinella prunicolor *	REH9880	Australia	MH614676	MH614725	MH614771	MH614840	Vadthanarat et al. 2019
* Fistulinella prunicolor *	REH9502	Australia	MG212544	MG212588	MG212630	–	Vadthanarat et al. 2018
* Gymnogaster boletoides *	NY01194009	Australia	–	KT990768	KT990406	–	Wu et al. 2016
* Harrya atriceps *	REH7403	Costa Rica	–	JX889702	–	–	Halling et al. 2012b
* Harrya chromapes *	HKAS50527	China	–	KF112270	KF112792	–	Wu et al. 2014
* Harrya chromapes *	HKAS49416	China	HQ326840	HQ326863	–	–	Li et al. 2011
* Harrya moniliformis *	HKAS49627	China	–	KT990881	KT990500	–	Wu et al. 2016
Heimioporus cf. mandarinus	OR0661	Thailand	MG212545	MG212589	MG212631	MH614841*	Vadthanarat et al. 2018; *Vadthanarat et al. 2019
* Heimioporus japonicus *	OR0114	Thailand	KT823971	KT824037	KT824004	MH614842*	Raspé et al. 2016; *Vadthanarat et al. 2019
* Heimioporus retisporus *	HKAS52237	China	–	KF112228	KF112806	–	Wu et al. 2014
*Heimioporus* sp.	OR0218	Thailand	MG212546	MG212590	MG212632	–	Vadthanarat et al. 2018
* Hemileccinum depilatum *	AF2845	Belgium	MG212547	MG212591	MG212633	MH614843*	Vadthanarat et al. 2018; *Vadthanarat et al. 2019
* Hemileccinum impolitum *	ADK4078	Belgium	MG212548	MG212592	MG212634	MH614844*	Vadthanarat et al. 2018; *Vadthanarat et al. 2019
* Hemileccinum indecorum *	OR0863	Thailand	MH614677	MH614726	MH614772	MH614845	Vadthanarat et al. 2019
* Hemileccinum rugosum *	HKAS84970	China	–	KT990773	KT990412	–	Wu et al. 2016
* Hortiboletus amygdalinus *	HKAS54166	China	–	KT990777	KT990416	–	Wu et al. 2016
* Hortiboletus rubellus *	VDKO0403	Belgium	MH614679	–	MH614774	MH614847	*Vadthanarat et al. 2019
*Hortiboletus* sp.	HKAS51239	China	–	KF112184	KF112695	–	Wu et al. 2014
*Hortiboletus* sp.	HKAS50466	China	–	KF112183	KF112694	–	Wu et al. 2014
*Hortiboletus* sp.	HKAS51292	China	–	KF112181	KF112692	–	Wu et al. 2014
*Hortiboletus* sp.	HKAS76673	China	–	KF112182	KF112693	–	Wu et al. 2014
* Hortiboletus subpaludosus *	HKAS59608	China	–	KF112185	KF112696	–	Wu et al. 2014
Hourangia cf. pumila	OR0762	Thailand	MH614680	MH614728	MH614775	MH614848	Vadthanarat et al. 2019
* Hourangia cheoi *	HKAS74744	China	–	KF112285	KF112772	–	Wu et al. 2014
* Hourangia cheoi *	Zhu108	China	–	KP136979	KP136928	–	Zhu et al. 2015
* Hourangia nigropunctata *	HKAS 57427	China	–	KP136927	KP136978	–	Zhu et al. 2015
* Hymenoboletus luteopurpureus *	HKAS46334	China	–	KF112271	KF112795	–	Wu et al. 2014
* Imleria badia *	VDKO0709	Belgium	KT823983	KT824049	KT824016	MH614849*	Raspé et al. 2016; *Vadthanarat et al. 2019
* Imleria obscurebrunnea *	OR0263	China	MH614681	MH614729	MH614776	MH614850	Vadthanarat et al. 2019
* Imleria subalpina *	HKAS74712	China	–	KF112189	KF112706	–	Wu et al. 2014
* Lanmaoa angustispora *	HKAS74759	China	–	KM605155	KM605178	–	Wu et al. 2015
* Lanmaoa angustispora *	HKAS74765	China	–	KF112159	KF112680	–	Wu et al. 2014
* Lanmaoa asiatica *	HKAS54094	China	–	KF112161	KF112682	–	Wu et al. 2014
* Lanmaoa asiatica *	HKAS63603	China	–	KM605153	KM605176	–	Wu et al. 2015
* Lanmaoa asiatica *	OR0228	China	MH614682	MH614730	MH614777	MH614851	Vadthanarat et al. 2019
* Lanmaoa carminipes *	BOTH4591	USA	MG897419	MG897429	MG897439	MH614852*	Phookamsak et al. 2019, *Vadthanarat et al. 2019
* Lanmaoa flavorubra *	NY775777	Costa Rica	–	KF112160	KF112681	–	Wu et al. 2014
* Lanmaoa pallidorosea *	BOTH4432	USA	MG897417	MG897427	MG897437	MH614853*	Phookamsak et al. 2019, *Vadthanarat et al. 2019
*Lanmaoa* sp.	HKAS52518	China	–	KF112162	KF112683	–	Wu et al. 2014
*Lanmaoa* sp.	OR0130	Thailand	MH614683	MH614731	MH614778	MH614854	Vadthanarat et al. 2019
*Lanmaoa* sp.	OR0370	Thailand	MH614684	MH614732	MH614779	MH614855	Vadthanarat et al. 2019
Leccinellum aff. crocipodium	HKAS76658	China	–	KF112252	KF112728	–	Wu et al. 2014
Leccinellum aff. griseum	KPM-NC-0017832	Japan	KC552164	JN378450*	–	–	unpublished, *Orihara et al. 2012
* Leccinellum corsicum *	Buf4507	USA	–	KF030435	–	–	Nuhn et al. 2013
* Leccinellum cremeum *	HKAS90639	China	–	KT990781	KT990420	–	Wu et al. 2016
* Leccinellum crocipodium *	VDKO1006	Belgium	KT823988	KT824054	KT824021	MH614856*	Raspé et al. 2016; *Vadthanarat et al. 2019
*Leccinellum* sp.	KPM-NC-0018041	Japan	KC552165	KC552094	–	–	Orihara et al. 2016
*Leccinellum* sp.	OR0711	Thailand	MH614685	MH614733	MH614780	–	Vadthanarat et al. 2019
* Leccinum monticola *	HKAS76669	China	–	KF112249	KF112723	–	Wu et al. 2014
* Leccinum quercinum *	HKAS63502	China	–	KF112250	KF112724	–	Wu et al. 2014
* Leccinum scabrum *	RW105a	Belgium	KT823979	KT824045	KT824012	MH614857*	Raspé et al. 2016; *Vadthanarat et al. 2019
* Leccinum scabrum *	VDKO0938	Belgium	MG212549	MG212593	MG212635	MH614858*	Vadthanarat et al. 2018; *Vadthanarat et al. 2019
* Leccinum scabrum *	KPM-NC-0017840	Scotland	KC552170	JN378455	–	–	Orihara et al. 2016; Orihara et al. 2012
* Leccinum schistophilum *	VDKO1128	Belgium	KT823989	KT824055	KT824022	MH614859*	Raspé et al. 2016; *Vadthanarat et al. 2019
* Leccinum variicolor *	VDKO0844	Belgium	MG212550	MG212594	MG212636	MH614860*	Vadthanarat et al. 2018; *Vadthanarat et al. 2019
* Mucilopilus castaneiceps *	HKAS75045	China	–	KF112211	KF112735	–	Wu et al. 2014
* Neoboletus brunneissimus *	HKAS52660	China	–	KF112143	KF112650	–	Wu et al. 2014
* Neoboletus brunneissimus *	HKAS57451	China	–	KM605149	KM605172	–	Wu et al. 2015
* Neoboletus brunneissimus *	OR0249	China	MG212551	MG212595	MG212637	MH614861*	Vadthanarat et al. 2018; *Vadthanarat et al. 2019
* Neoboletus hainanensis *	HKAS59469	China	–	KF112175	KF112669	–	Wu et al. 2014
* Neoboletus junquilleus *	AF2922	France	MG212552	MG212596	MG212638	MH614862*	Vadthanarat et al. 2018; *Vadthanarat et al. 2019
* Neoboletus magnificus *	HKAS54096	China	–	KF112149	KF112654	–	Wu et al. 2014
* Neoboletus magnificus *	HKAS74939	China	–	KF112148	KF112653	–	Wu et al. 2014
* Neoboletus sanguineoides *	HKAS55440	China	–	KF112145	KF112652	–	Wu et al. 2014
*Neoboletus* sp.	HKAS76851	China	–	KF112144	KF112651	–	Wu et al. 2014
*Neoboletus* sp.	OR0128	Thailand	MH614686	MH614734	MH614781	MH614863	Vadthanarat et al. 2019
* Neoboletus tomentulosus *	HKAS53369	China	–	KF112154	KF112659	–	Wu et al. 2014
* Neoboletus erythropus *	VDKO0690	Belgium	KT823982	KT824048	KT824015	MH614864*	Raspé et al. 2016; *Vadthanarat et al. 2019
* Octaviania asahimontana *	KPM-NC-17824	Japan	KC552154	JN378430	–	–	Orihara et al. 2016; Orihara et al. 2012
* Octaviania asterosperma *	AQUI3899	Italy	KC552159	KC552093	–	–	Orihara et al. 2016
* Octaviania celatifilia *	KPM-NC-17776	Japan	KC552147	JN378416	–	–	Orihara et al. 2016; Orihara et al. 2012
* Octaviania cyanescens *	PNW-FUNGI-5603	USA	KC552160	JN378438	–	–	Orihara et al. 2016; Orihara et al. 2012
* Octaviania decimae *	KPM-NC17763	Japan	KC552145	JN378409	–	–	Orihara et al. 2016; Orihara et al. 2012
* Octaviania tasmanica *	MEL2128484	Australia	KC552157	JN378437	–	–	Orihara et al. 2016; Orihara et al. 2012
* Octaviania tasmanica *	MEL2341996	Australia	KC552156	JN378436	–	–	Orihara et al. 2016; Orihara et al. 2012
* Octaviania zelleri *	MES270	USA	KC552161	JN378440	–	–	Orihara et al. 2016; Orihara et al. 2012
* Parvixerocomus pseudoaokii *	OR0155	China	MG212553	MG212597	MG212639	MH614865	Vadthanarat et al. 2019
* Phylloporus bellus *	OR0473	China	MH580778	MH580798	MH580818	MH614866*	Chuankid et al. 2019; *Vadthanarat et al. 2019
* Phylloporus brunneiceps *	OR0050	Thailand	KT823968	KT824034	KT824001	MH614867*	Raspé et al. 2016; *Vadthanarat et al. 2019
* Phylloporus castanopsidis *	OR0052	Thailand	KT823969	KT824035	KT824002	MH614868*	Raspé et al. 2016; *Vadthanarat et al. 2019
* Phylloporus imbricatus *	HKAS68642	China	–	KF112299	KF112786	–	Wu et al. 2014
* Phylloporus luxiensis *	HKAS75077	China	–	KF112298	KF112785	–	Wu et al. 2014
* Phylloporus maculatus *	OR0285	China	MH580780	MH580800	MH580820	–	Chuankid et al. 2019
* Phylloporus pelletieri *	WU18746	Austria	MH580781	MH580801	MH580821	MH614869*	Chuankid et al. 2019; *Vadthanarat et al. 2019
* Phylloporus pusillus *	OR1158	Thailand	MH580783	MH580803	MH580823	MH614870*	Chuankid et al. 2019; *Vadthanarat et al. 2019
* Phylloporus rhodoxanthus *	WU17978	USA	MH580785	MH580805	MH580824	MH614871*	Chuankid et al. 2019; *Vadthanarat et al. 2019
* Phylloporus rubeolus *	OR0251	China	MH580786	MH580806	MH580825	MH614872*	Chuankid et al. 2019; *Vadthanarat et al. 2019
* Phylloporus rubiginosus *	OR0169	China	MH580788	MH580808	MH580827	MH614873*	Chuankid et al. 2019; *Vadthanarat et al. 2019
*Phylloporus* sp.	OR0896	Thailand	MH580790	MH580810	MH580829	MH614874*	Chuankid et al. 2019; *Vadthanarat et al. 2019
* Phylloporus subbacillisporus *	OR0436	China	MH580792	MH580812	MH580831	MH614875*	Chuankid et al. 2019; *Vadthanarat et al. 2019
* Phylloporus subrubeolus *	BC022	Thailand	MH580793	MH580813	MH580832	MH614876*	Chuankid et al. 2019; *Vadthanarat et al. 2019
* Phylloporus yunnanensis *	OR0448	China	MG212554	MG212598	MG212640	MH614877*	Vadthanarat et al. 2018; *Vadthanarat et al. 2019
* Porphyrellus castaneus *	OR0241	China	MG212555	MG212599	MG212641	MH614878*	Vadthanarat et al. 2018; *Vadthanarat et al. 2019
Porphyrellus cf. nigropurpureus	ADK3733	Benin	MH614687	MH614735	MH614782	MH614879	Vadthanarat et al. 2019
* Porphyrellus nigropurpureus *	HKAS74938	China	–	KF112246	KF112763	–	Wu et al. 2014
* Porphyrellus porphyrosporus *	MB97-023	Germany	DQ534609	GU187734	GU187800	–	Binder & Hibbett 2006; Binder et al. 2010
*Porphyrellus* sp.	HKAS53366	China	–	KF112241	KF112716	–	Wu et al. 2014
*Porphyrellus* sp.	JD659	Burundi	MH614688	MH614736	MH614783	MH614880	Vadthanarat et al. 2019
*Porphyrellus* sp.	OR0222	Thailand	MH614689	MH614737	MH614784	MH614881	Vadthanarat et al. 2019
Pulveroboletus aff. ravenelii	ADK4360	Togo	KT823957	KT824023	KT823990	MH614882*	Raspé et al. 2016; *Vadthanarat et al. 2019
Pulveroboletus aff. ravenelii	ADK4650	Togo	KT823959	KT824025	KT823992	MH614883*	Raspé et al. 2016; *Vadthanarat et al. 2019
Pulveroboletus aff. ravenelii	HKAS53351	China	–	KF112261	KF112712	–	Wu et al. 2014
* Pulveroboletus fragrans *	OR0673	Thailand	KT823977	KT824043	KT824010	MH614884*	Raspé et al. 2016; *Vadthanarat et al. 2019
* Pulveroboletus ravenelii *	REH2565	USA	KU665635	KU665636	KU665637	MH614885*	Raspé et al. 2016; *Vadthanarat et al. 2019
*Pulveroboletus* sp.	HKAS74933	China	–	KF112262	KF112713	–	Wu et al. 2014
Retiboletus aff. nigerrimus	OR0049	Thailand	KT823967	KT824033	KT824000	MH614886*	Raspé et al. 2016; *Vadthanarat et al. 2019
* Retiboletus brunneolus *	HKAS52680	China	–	KF112179	KF112690	–	Wu et al. 2014
* Retiboletus fuscus *	HKAS59460	China	–	JQ928580	JQ928601	–	Hosen et al. 2013
* Retiboletus fuscus *	OR0231	China	MG212556	MG212600	MG212642	MH614887*	Vadthanarat et al. 2018; *Vadthanarat et al. 2019
* Retiboletus griseus *	MB03-079	USA	KT823964	KT824030	KT823997	MH614888*	Raspé et al. 2016; *Vadthanarat et al. 2019
* Retiboletus kauffmanii *	OR0278	China	MG212557	MG212601	MG212643	MH614889*	Vadthanarat et al. 2018; *Vadthanarat et al. 2019
* Retiboletus nigerrimus *	HKAS53418	China	–	KT990824	KT990462	–	Wu et al. 2016
* Retiboletus sinensis *	HKAS59832	China	–	KT990827	KT990464	–	Wu et al. 2016
* Retiboletus zhangfeii *	HKAS59699	China	–	JQ928582	JQ928603	–	Hosen et al. 2013
* Rhodactina himalayensis *	CMU25117	Thailand	MG212558	MG212602, MG212603	–	–	Vadthanarat et al. 2018
* Rhodactina rostratispora *	SV170	Thailand	MG212560	MG212605	MG212645	–	Vadthanarat et al. 2018
* Rossbeevera cryptocyanea *	KPM-NC17843	Japan	KT581441	KC552072	–	–	Orihara et al. 2016
* Rossbeevera eucyanea *	TNS-F-36986	Japan	KC552115	KC552068	–	–	Orihara et al. 2016
* Rossbeevera griseovelutina *	TNS-F-36989	Japan	KC552124	KC552076	–	–	Orihara et al. 2016
* Rossbeevera pachydermis *	KPM-NC23336	New Zealand	KJ001064	KP222912	–	–	Orihara et al. 2016
* Rossbeevera vittatispora *	OSC61484	Australia	KC552109	JN378446	–	–	Orihara et al. 2016; Orihara et al. 2012
* Royoungia reticulata *	HKAS52253	China	–	KT990786	KT990427	–	Wu et al. 2016
* Royoungia rubina *	HKAS53379	China	–	KF112274	KF112796	–	Wu et al. 2014
* Rubroboletus latisporus *	HKAS80358	China	–	KP055020	KP055029	–	Zhao et al. 2014b
* Rubroboletus legaliae *	VDKO0936	Belgium	KT823985	KT824051	KT824018	MH614890*	Raspé et al. 2016; *Vadthanarat et al. 2019
* Rubroboletus rhodosanguineus *	BOTH4263	USA	MG897416	MG897426	MG897436	MH614891*	Phookamsak et al. 2019, *Vadthanarat et al. 2019
* Rubroboletus rhodoxanthus *	HKAS84879	Germany	–	KT990831	KT990468	–	Wu et al. 2016
* Rubroboletus satanas *	VDKO0968	Belgium	KT823986	KT824052	KT824019	MH614892*	Raspé et al. 2016; *Vadthanarat et al. 2019
* Rubroboletus sinicus *	HKAS68620	China	–	KF112146	KF112661	–	Wu et al. 2014
*Rubroboletus* sp.	HKAS68679	China	–	KF112147	KF112662	–	Wu et al. 2014
* Rugiboletus brunneiporus *	HKAS68586	China	–	KF112197	KF112719	–	Wu et al. 2014
* Rugiboletus brunneiporus *	HKAS83209	China	–	KM605144	KM605168	–	Wu et al. 2015
* Rugiboletus extremiorientalis *	HKAS63635	China	–	KF112198	KF112720	–	Wu et al. 2014
* Rugiboletus extremiorientalis *	HKAS76663	China	–	KM605147	KM605170	–	Wu et al. 2015
* Rugiboletus extremiorientalis *	OR0406	Thailand	MG212562	MG212607	MG212647	MH614893*	Vadthanarat et al. 2018; *Vadthanarat et al. 2019
*Rugiboletus* sp.	HKAS55373	China	–	KF112303	KF112804	–	Wu et al. 2014
* Singerocomus inundabilis *	TWH9199	Guyana	MH645588	MH645596	LC043089*	MH645609	*Henkel et al. 2016; Vadthanarat et al. 2019
* Singerocomus rubriflavus *	TWH9585	Guyana	MH645589	MH645597	–	MH645610	Vadthanarat et al. 2019
* Spongiforma thailandica *	DED7873	Thailand	MG212563	KF030436*	MG212648	MH614894**	*Nuhn et al. 2013; Vadthanarat et al. 2018; **Vadthanarat et al. 2019
* Strobilomyces atrosquamosus *	HKAS55368	China	–	KT990839	KT990476	–	Wu et al. 2016
* Strobilomyces echinocephalus *	OR0243	China	MG212564	MG212608	MG212649	–	Vadthanarat et al. 2018
* Strobilomyces strobilaceus *	RW103	Belgium	KT823978	KT824044	KT824011	MH614895*	Raspé et al. 2016; *Vadthanarat et al. 2019
* Strobilomyces strobilaceus *	MB-03-102	USA	DQ534607*	AY883428	AY786065	–	Binder and Hibbett 2006*, Unpublished
* Strobilomyces mirandus *	OR0115	Thailand	KT823972	KT824038	KT824005	MH614896*	Raspé et al. 2016; *Vadthanarat et al. 2019
*Strobilomyces* sp.	OR0259	China	MG212565	MG212609	MG212650	MH614897*	Vadthanarat et al. 2018; *Vadthanarat et al. 2019
*Strobilomyces* sp.	OR0778	Thailand	MG212566	MG212610	MG212651	MH614899*	Vadthanarat et al. 2018; *Vadthanarat et al. 2019
*Strobilomyces* sp.	OR0319	Thailand	MH614690	MH614738	MH614785	MH614898	Vadthanarat et al. 2019
*Strobilomyces* sp.	OR1092	Thailand	MH614691	MH614739	MH614786	MH614900	Vadthanarat et al. 2019
* Strobilomyces verruculosus *	HKAS55389	China	–	KF112259	KF112813	–	Wu et al. 2014
* Suillellus amygdalinus *	112605ba	USA	–	JQ327024	–	–	Halling et al. 2012a
* Suillellus luridus *	VDKO0241b	Belgium	KT823981	KT824047	KT824014	MH614901*	Raspé et al. 2016; *Vadthanarat et al. 2019
* Suillellus queletii *	VDKO1185	Belgium	MH645590	MH645598	MH645604	MH645611	Vadthanarat et al. 2019
* Suillellus subamygdalinus *	HKAS57262	China	–	KF112174	KF112660	–	Wu et al. 2014
* Sutorius australiensis *	REH9441	Australia	MG212567	JQ327032*	MG212652	–	*Halling et al. 2012a; Vadthanarat et al. 2018
* Sutorius eximius *	REH9400	USA	MG212568	JQ327029*	MG212653	MH614902**	*Halling et al. 2012a; Vadthanarat et al. 2018; **Vadthanarat et al. 2019
* Sutorius ferrugineus *	HKAS77718	China	–	KT990789	KT990431	–	Wu et al. 2016
* Sutorius flavidus *	HKAS59443	China	–	KU974136	KU974144	–	Wu et al. 2016
* Sutorius rubriporus *	HKAS83026	China	–	KT990795	KT990437	–	Wu et al. 2016
* Sutorius sanguineus *	HKAS80823	China	–	KT990802	KT990442	–	Wu et al. 2016
*Sutorius* sp.	OR0378B	Thailand	MH614692	MH614740	MH614787	MH614903	Vadthanarat et al. 2019
*Sutorius* sp.	OR0379	Thailand	MH614693	MH614741	MH614788	MH614904	Vadthanarat et al. 2019
* Tengioboletus glutinosus *	HKAS53425	China	–	KF112204	KF112800	–	Wu et al. 2014
* Tengioboletus reticulatus *	HKAS53426	China	–	KF112313	KF112828	–	Wu et al. 2014
*Tengioboletus* sp.	HKAS76661	China	–	KF112205	KF112801	–	Wu et al. 2014
* Turmalinea persicina *	KPM-NC18001	Japan	KC552130	KC552082	–	–	Orihara et al. 2016
* Turmalinea yuwanensis *	KPM-NC18011	Japan	KC552138	KC552089	–	–	Orihara et al. 2016
* Tylocinum griseolum *	HKAS50281	China	–	KF112284	KF112730	–	Wu et al. 2014
* Tylopilus alpinus *	HKAS55438	China	–	KF112191	KF112687	–	Wu et al. 2014
* Tylopilus atripurpureus *	HKAS50208	China	–	KF112283	KF112799	–	Wu et al. 2014
*Tylopilusballoui* s.l.	OR0039	Thailand	KT823965	KT824031	KT823998	MH614905*	Raspé et al. 2016; *Vadthanarat et al. 2019
* Tylopilus brunneirubens *	HKAS53388	China	–	KF112192	KF112688	–	Wu et al. 2014
* Tylopilus felleus *	VDKO0992	Belgium	KT823987	KT824053	KT824020	MH614906*	Raspé et al. 2016; *Vadthanarat et al. 2019
* Tylopilus ferrugineus *	BOTH3639	USA	MH614694	MH614742	MH614789	MH614907	Vadthanarat et al. 2019
* Tylopilus otsuensis *	HKAS53401	China	–	KF112224	KF112797	–	Wu et al. 2014
*Tylopilus* sp.	HKAS74925	China	–	KF112222	KF112739	–	Wu et al. 2014
*Tylopilus* sp.	HKAS50229	China	–	KF112216	KF112769	–	Wu et al. 2014
*Tylopilus* sp.	JD598	Gabon	MH614695	MH614743	MH614790	MH614908	Vadthanarat et al. 2019
*Tylopilus* sp.	OR0252	China	MG212569	MG212611	MG212654	MH614909*	Vadthanarat et al. 2018; *Vadthanarat et al. 2019
*Tylopilus* sp.	OR0542	Thailand	MG212570	MG212612	MG212655	MH614910*	Vadthanarat et al. 2018; *Vadthanarat et al. 2019
*Tylopilus* sp.	OR0583	Thailand	MH614696	MH614744	–	–	Vadthanarat et al. 2019
*Tylopilus* sp.	OR1009	Thailand	MH614697	–	MH614791	MH614911	Vadthanarat et al. 2019
* Tylopilus vinaceipallidus *	HKAS50210	China	–	KF112221	KF112738	–	Wu et al. 2014
* Tylopilus vinaceipallidus *	OR0137	China	MG212571	MG212613	MG212656	MH614912*	Vadthanarat et al. 2018; *Vadthanarat et al. 2019
* Tylopilus violaceobrunneus *	HKAS89443	China	–	KT990886	KT990504	–	Wu et al. 2016
* Tylopilus virens *	KPM-NC-0018054	Japan	KC552174	KC552103	–	–	Unpublished
* Veloporphyrellus alpinus *	HKAS68301	China	JX984515	JX984550	–	–	Li et al. 2014
* Veloporphyrellus conicus *	REH8510	Belize	MH614698	MH614745	MH614792	MH614913	Vadthanarat et al. 2019
* Veloporphyrellus gracilioides *	HKAS53590	China	–	KF112210	KF112734	–	Wu et al. 2014
* Veloporphyrellus pseudovelatus *	HKAS59444	China	JX984519	JX984553	–	–	Li et al. 2014
* Veloporphyrellus velatus *	HKAS63668	China	JX984523	JX984554	–	–	Li et al. 2014
* Xanthoconium affine *	NY00815399	USA	–	KT990850	KT990486	–	Wu et al. 2016
* Xanthoconium porophyllum *	HKAS90217	China	–	KT990851	KT990487	–	Wu et al. 2016
* Xanthoconium sinense *	HKAS77651	China	–	KT990853	KT990488	–	Wu et al. 2016
* Xerocomellus chrysenteron *	VDKO0821	Belgium	KT823984	KT824050	KT824017	MH614914*	Raspé et al. 2016; *Vadthanarat et al. 2019
* Xerocomellus cisalpinus *	ADK4864	Belgium	KT823960	KT824026	KT823993	MH614915*	Raspé et al. 2016; *Vadthanarat et al. 2019
* Xerocomellus communis *	HKAS50467	China	–	KT990858	KT990494	–	Wu et al. 2016
* Xerocomellus corneri *	HKAS90206	Philippines	–	KT990857	KT990493	–	Wu et al. 2016
* Xerocomellus porosporus *	VDKO0311	Belgium	MH614678	MH614727	MH614773	MH614846	Vadthanarat et al. 2019
* Xerocomellus ripariellus *	VDKO0404	Belgium	MH614699	MH614746	MH614793	MH614916	Vadthanarat et al. 2019
*Xerocomellus* sp.	HKAS56311	China	–	KF112170	KF112684	–	Wu et al. 2014
Xerocomus aff. macrobbii	HKAS56280	China	–	KF112265	KF112708	–	Wu et al. 2014
* Xerocomus fulvipes *	HKAS76666	China	–	KF112292	KF112789	–	Wu et al. 2014
* Xerocomus magniporus *	HKAS58000	China	–	KF112293	KF112781	–	Wu et al. 2014
*Xerocomus* s.s. sp.	OR0237	China	MH580796	MH580816	MH580835	–	Chuankid et al. 2019
*Xerocomus* s.s. sp.	OR0443	China	MH580797	MH580817	MH580836	MH614917*	Chuankid et al. 2019; *Vadthanarat et al. 2019
*Xerocomus* sp.	OR0053	Thailand	MH580795	MH580815	MH580834	MH614918*	Chuankid et al. 2019; *Vadthanarat et al. 2019
* Xerocomus subtomentosus *	VDKO0987	Belgium	MG212572	MG212614	MG212657	MH614919*	Vadthanarat et al. 2018; *Vadthanarat et al. 2019
* Zangia citrina *	HKAS52684	China	HQ326850	HQ326872	–	–	Li et al. 2011
* Zangia olivacea *	HKAS45445	China	HQ326854	HQ326873	–	–	Li et al. 2011
* Zangia olivaceobrunnea *	HKAS52272	China	HQ326857	HQ326876	–	–	Li et al. 2011
* Zangia roseola *	HKAS75046	China	–	KF112269	KF112791	–	Wu et al. 2014
* Zangia roseola *	HKAS51137	China	HQ326858	HQ326877	–	–	Li et al. 2011

### Taxonomy

#### Erythrophylloporus

Taxon classificationFungiBoletalesBoletaceae

Ming Zhang & T.H. Li, Mycosystema 37(9): 1111–1126 (2018)

##### Description.

*Basidiomata* stipitate-pileate with lamellate hymenophore, small to medium-sized; *Pileus* subhemispheric to convex when young becoming convex to plano-convex to plano-subdepressed when old, dry, pruinose or velutinous, subtomentose to tomentose, yellowish-orange to red; *pileus context* vivid yellow to yellowish-orange. *Hymenophore* lamellae, slightly thick, decurrent, deeply yellowish-orange to deep orange or reddish-orange to orange red or brownish-orange to red. *Stipe* central to slightly excentric, cylindrical or clavate, yellowish- to reddish-orange to yellowish red, with scattered yellowish- to reddish-orange to red scales on surface, with bright yellow basal mycelium; *stipe context* solid, yellow to reddish-yellow or yellow with olivaceous brown. *Staining* none or slightly reddening or greening or gradually bluing or dark violet, greyish to blackish-blue when bruised on the basidiomata or context or lamellae. *Spore print* olivaceous brown. *Basidiospores* ovoid or ellipsoid to broadly ellipsoid to subovoid, thin-walled, with non-bacillate surface. *Basidia* clavate to narrowly clavate. *Cheilocystidia and pleurocystidia* present, subcylindrical or narrowly conical to narrowly fusiform to ventricose with slightly or obtuse apex, thin-walled, sometimes thick-walled, originating more or less deeply in the sub hymenium or from hymenophoral trama, hyaline or sometimes containing yellowish-brown pigments. *Pileipellis* a subcutis to cutis to trichoderm to palisadoderm, composed of thin to slightly thick-walled hyphae. *Clamp connection* absent in all tissues.

##### Typus species.

*Erythrophylloporuscinnabarinus* Ming Zhang & T.H. Li.

##### Known Distribution.

Asia (China and Thailand), North America (Mexico) and Central America (Costa Rica).

##### Remarks.

*Erythrophylloporus* is easily distinguished from other lamellate Boletaceae genera by a combination of the following characters: the intense orange to red colour of the pileus and lamellae; bright yellow basal mycelium; ovoid or ellipsoid to broadly ellipsoid to subovoid basidiospores with non-bacillate surface; pleurocystidia originating more or less deeply in the subhymenium or from hymenophoral trama.

#### Erythrophylloporus paucicarpus

Taxon classificationFungiBoletalesBoletaceae

Raspé, Vadthanarat & Lumyong sp. nov.

823605

**Figure 2.**
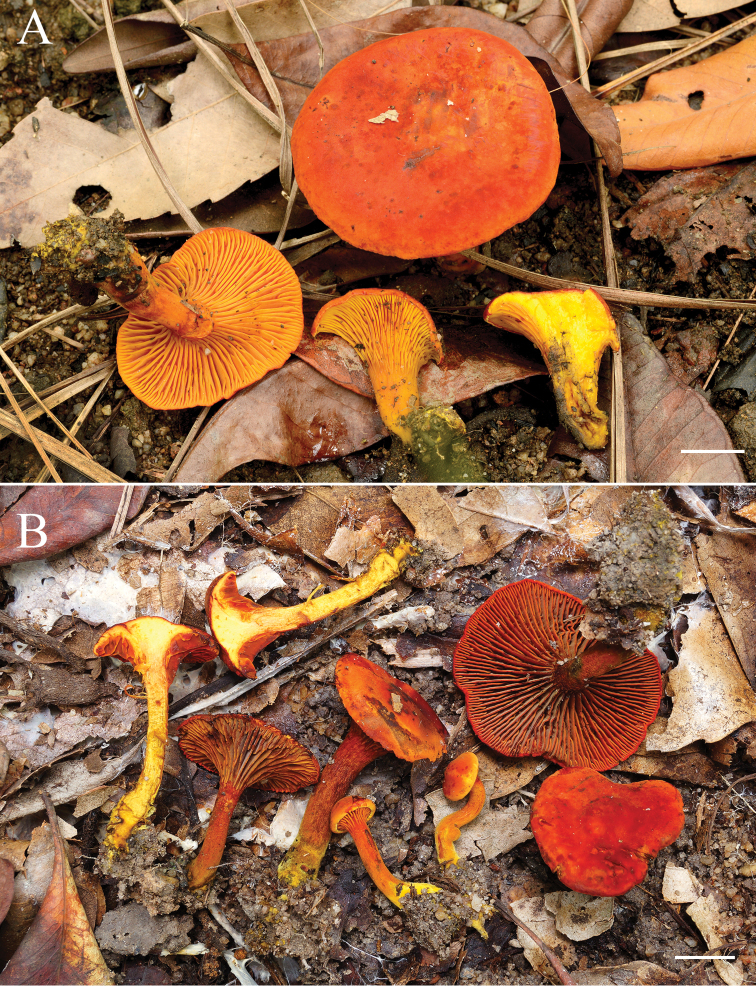
Habits of Thai *Erythrophylloporus* species **A***E.paucicarpus***B***E.suthepensis*. Scale bars: 1 cm.

**Figure 3.**
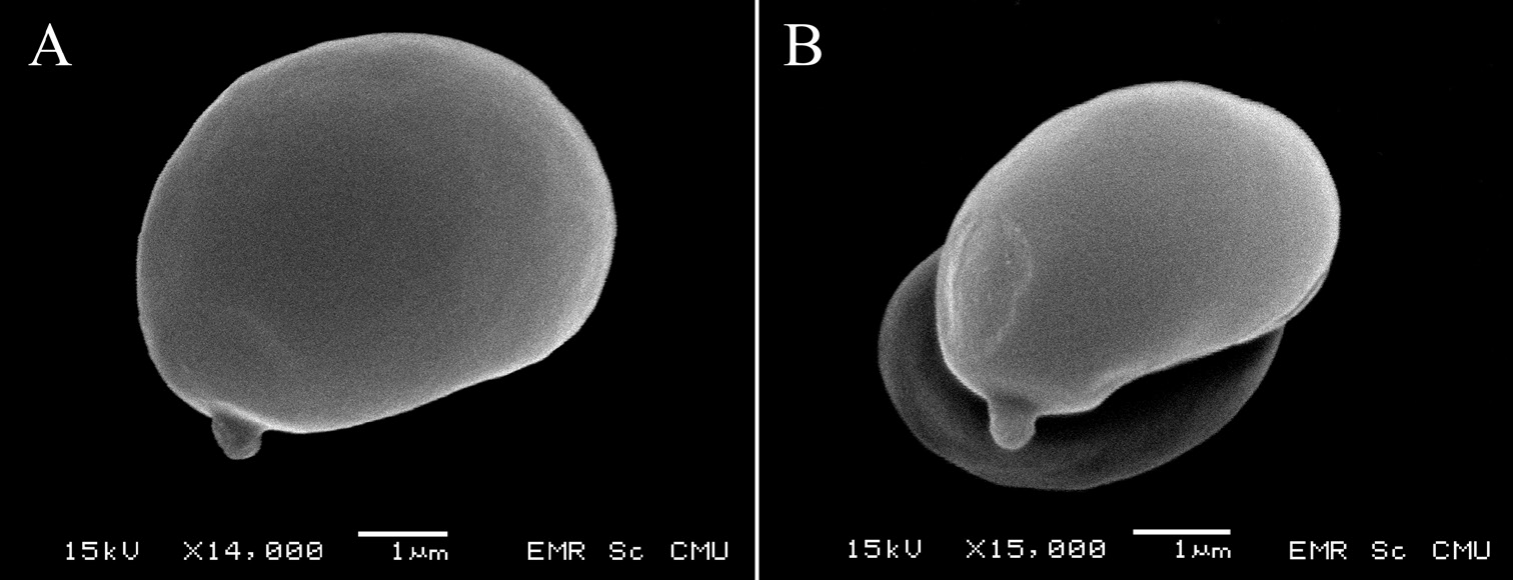
Scanning electron micrographs of basidiospores from Thai *Erythrophylloporus* show smooth surfaces **A***E.paucicarpus***B***E.suthepensis*. Scale bars: 1 µm.

**Figure 4.**
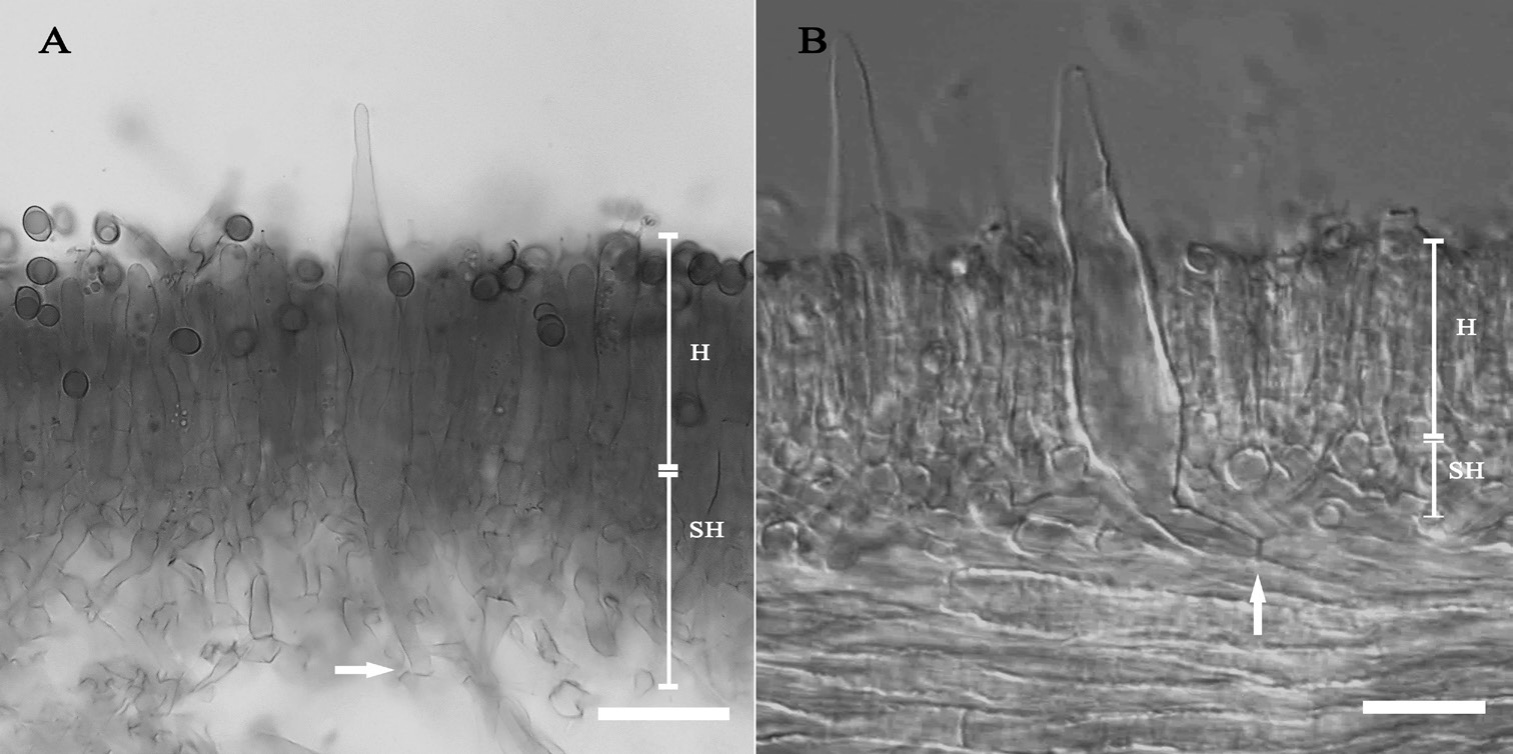
Origin of pleurocystidia (white arrow), more or less deep in the subhymenium or from hymenophoral trama **A***E.paucicarpus***B***E.suthepensis* – hymenium (H), subhymenium (SH), Scale bars: 25 µm (**A–B**).

**Figure 5.**
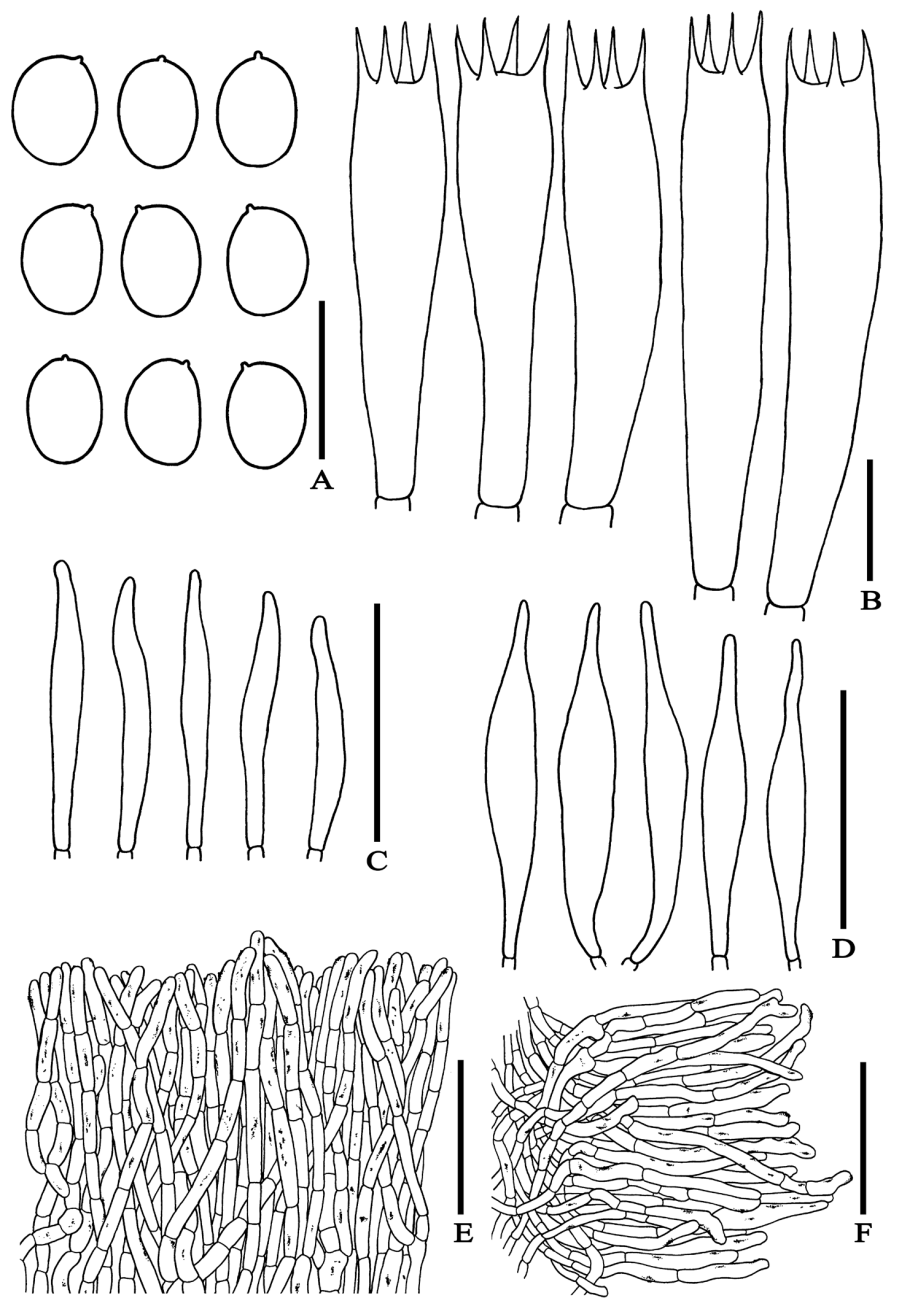
Microscopic features of *Erythrophylloporuspaucicarpus***A** basidiospores **B** basidia **C** cheilocystidia **D** pleurocystidia **E** pileipellis **F** stipitipellis. – Scale bars: 10 µm (**A–B**); 50 µm (**C–F**). All drawings were made from the type (OR1151).

Figs 2A , 3A , 4A and 5

##### Holotype.

THAILAND, Chiang Mai Province, Mae On District, Huay Kaew, 18°52'0"N, 99°17'30"E, elev. 700 m, 16 August 2016, *O. Raspé & S. Vadthanarat*, OR1151, (holotype: CMUB, isotype: BR).

##### Etymology.

from Latin “pauci-” meaning few and “carpus” meaning fruits or what is harvested, refers to the low number of basidiomata produced.

##### Description.

*Basidiomata* stipitate-pileate with lamellate hymenophore, small to medium-sized. *Pileus* 2.3–5.5 cm in diameter, plano-convex with involute margin at first becoming almost plane to slightly depressed with inflexed to straight margin, irregularly and coarsely crenate in age, sometimes with low and broad umbo and a few to several verrucae, especially when young; *surface* more or less even, tomentose, dull, slightly moist, colour distribution patchy with red to brownish-orange (9B8 to 9C8), brownish-red (10E8 to 10D8) becoming orange-red to orange (8B/C8 to 6B7) at the margin when old, abruptly paler at the margin. *Pileus context* 3–4 mm thick half-way to the margin, tough, colour distribution even, yellow (3A6) to yellowish-orange (4A5), slowly reddening when exposed, especially at the centre and above lamellae. *Stipe* 2.4–4.5 × 0.7–1.3 cm, central or sometimes slightly eccentric, clavate with strigose base, straight to curved, terete, even, dull, dry, tomentose, yellowish-orange (4–5A7–8) to orange (6–7A7–8) with orange to yellowish-orange (7B/C7–8 to 4A7–8) coarse scales, with bright yellow (2A6–7) basal mycelium. *Stipe context* solid, fleshy fibrous, yellow marbled with olivaceous brown (4D5, 5D5). *Hymenophore* lamellate; lamellae decurrent, close, thick, 40–42 lamellae, with 4–6 different lengths of lamellullae, 2–4.5 mm wide half-way to margin, somewhat anastomosing, especially near the stipe, yellowish-orange (4-5A6-7) with orange to red tinge, slightly reddening when bruised. *Odour* rubbery; *Taste* not recorded. *Spore print* olive-brown (4E7).

*Macrochemical reactions*. KOH on pileus and stipe surface deep red at first, then red-brown to brown, with pale orange aura on the pileus; brown on pileus context, dark red-brown on stipe context; brownish-orange on hymenophore. NH_4_OH on pileus first red, then orange; on pileus context bluing at first then with a greenish tinge; on stipe surface and context briefly bluing; no reaction on hymenophore.

*Basidiospores* [208/4/4] (5.9–)6.1–6.8–7.5(–8) × (4.1–)4.6–5.1–5.5(–6) µm, *Q* = (1.2–)1.23–1.33–1.48(–1.56); from the type (OR1151) (6–)6.3–6.8–7.5(–7.8) × (4.6–)4.8–5.2–5.5(–6) µm, *Q* = (1.2–)1.22–1.31–1.48(–1.56), *N* = 88, broadly ellipsoid to ellipsoid, smooth under light microscope and SEM, yellowish to pale brown in water, hyaline in 5% KOH, thin-walled, inamyloid. *Basidia* 4–spored, (37.8–)38–45.6–54.7(–54.8) × (6.2–)–6.3–8–9.5(–9.6) µm, narrowly clavate to subcylindrical, attenuated towards the base, clampless, hyaline to yellowish hyaline in water, Melzer’s reagent and 5% KOH; *sterigmata* up to 5.5 µm long. *Cheilocystidia* (35.4–)35.5–49.9–61.8(–61.9) × (3.9–)3.9–6–7.7(–7.7) µm, narrowly fusiform with obtuse apex, projecting up to 30 µm, thin-walled, smooth, yellowish hyaline in water, in 5% KOH and NH_4_OH, inamyloid. *Pleurocystidia* (66.9–)67.4–80.3–93.5(–94.7) × (8.8–)8.9–11.7–16.1(–16.2) µm, abundant, narrowly conical with obtuse, somewhat prolonged apex, projecting up to 32 µm, thin-walled, smooth, yellowish hyaline in water, in 5% KOH and NH_4_OH, arising more or less deeply in the subhymenium or from hymenophoral trama, inamyloid. *Hymenophoral trama* subregular near the pileus context becoming slightly divergent near the edge, 87–238 µm wide, widest near the pileus context then getting narrower when close to the edge, composed of clampless hyphae 4.5–8 µm wide, yellowish hyaline in water, hyaline in 5% KOH and NH_4_OH. *Pileipellis* a palisadoderm to trichoderm 83–165 µm thick, composed of slightly thick-walled, cylindrical hyphae, terminal cells 16–46 × 4–6.5 µm with rounded apex, hyaline or yellowish hyaline to yellowish-orange hyaline hyphae ornamented with scattered fine epiparietal encrustation when observed in water, hyaline to yellowish hyaline in 5% KOH and NH_4_OH, inamyloid. *Pileus trama* composed of slightly thick-walled, strongly interwoven hyphae, 4.5–8.5 µm wide, inamyloid. *Stipitipellis* a disrupted palisadoderm perpendicular to the stipe axis, 63–145 µm thick, composed of slightly thick-walled, slightly rough, cylindrical, yellow to yellowish-orange in water, yellowish hyaline hyphae in 5% KOH and NH_4_OH, terminal cells 13–57 × 3–8 µm, cylindrical to irregular hyphae with rounded to notched apex; wall covered by dispersed fine encrustations when observed in water. *Caulocystidia* not seen. *Stipe trama* composed of parallel hyphae, densely packed, 4–8.5 µm wide; hyphae wall covered by dispersed encrustations when observed in water. *Clamp connections* not seen in any tissue.

##### Habit and habitat.

On soil, mostly solitary in dipterocarp forest dominated by *Dipterocarpustuberculatus*, *D.obtusifolius*, *Shoreaobtusa*, *S.siamensis*, *Quercus* spp. and *Lithocarpus* spp.

##### Known distribution.

Currently known only from Chiang Mai Province, northern Thailand.

##### Additional specimens examined.

– THAILAND, Chiang Mai Province, Muang District, Doi Suthep-Pui National Park, 18°48'05"N–98°55'40"E, elev. 800 m, 17 May 2015, *O. Raspé*, OR0615A (CMUB, BKF, BR); Mae Taeng District, Baan Tapa, 19°08'29"N, 98°45'47"E, elev. 1035 m, 4 August 2015, *O. Raspé & A. Thawthong*, OR0689 (MFLU, BR); Mae On District, Huay Kaew, 18°52'12"N, 99°18'12"E, elev. 780 m, 15 August 2016, *O. Raspé & S. Vadthanarat*, OR1135 (CMUB, BR).

##### Remarks.

*E.paucicarpus* is characterised by the following combination of features: orange to brownish- to orange-red basidiomata, yellowish-orange lamellae that turn slightly red when bruised; pileus context yellow to yellowish-orange that slowly reddens when exposed and mostly occurring as solitary basidiomata.

In the inferred molecular phylogeny, *E.paucicarpus* clustered close to *E.suthepensis* and *E.cinnabarinus* (65% BS and 1 PP), but the two species are different from *E.paucicarpus* in that they have darker lamellae which are orange to orange red or brownish-orange. Moreover, spores of *E.paucicarpus* are wider and longer (5.9–8 × 4.1–6 µm) than those of *E.suthepensis* (4.6–5.9 × 3.5–4.5 µm) and, on average, longer than those of *E.cinnabarinus* (5.5–7 × 4.5–5.5 µm) (Zhang and Li 2018). *Erythrophylloporuspaucicarpus* also differs from both species by the slight reddening of the context and lamellae when exposed or bruised, whereas *E.suthepensis* context seems unchanging when exposed and lamellae turn blue when bruised. In *E.cinnabarinus*, the context slowly turns dark violet, blackish-blue to dark blue when exposed and lamellae turn greyish-blue, or greyish-green when bruised (Zhang and Li 2018).

*Erythrophylloporuspaucicarpus* is different from the two New World species by the reddening of the context, whereas in *E.fagicola*, it turns blue and, in *E.aurantiacus*, the colour remains unchanged when exposed. Moreover, *E.fagicola* has somewhat thick-walled (0.8–3.5 µm) pleurocystidia (Montoya and Bandala 2011), which are not found in *E.paucicarpus*. Although the basidiospores of *E.paucicarpus* and *E.aurantiacus* are similar in size (*E.aurantiacus* = 6.0–7.5 × 4–5.5 µm), they differ in shape, being more ovoid in *E.aurantiacus* than in *E.paucicarpus*. *Erythrophylloporuspaucicarpus* also differs from *E.aurantiacus* by macro-chemical reactions. In the latter, the pileus surface and pileus context are unchanging with NH_4_OH (Halling et al. 1999), while in *E.paucicarpus*, the pileus becomes orange to red and the pileus context initially turns blue then with a greenish tinge.

#### Erythrophylloporus suthepensis

Taxon classificationFungiBoletalesBoletaceae

Vadthanarat, Raspé & Lumyong sp. nov.

823606

**Figure 6.**
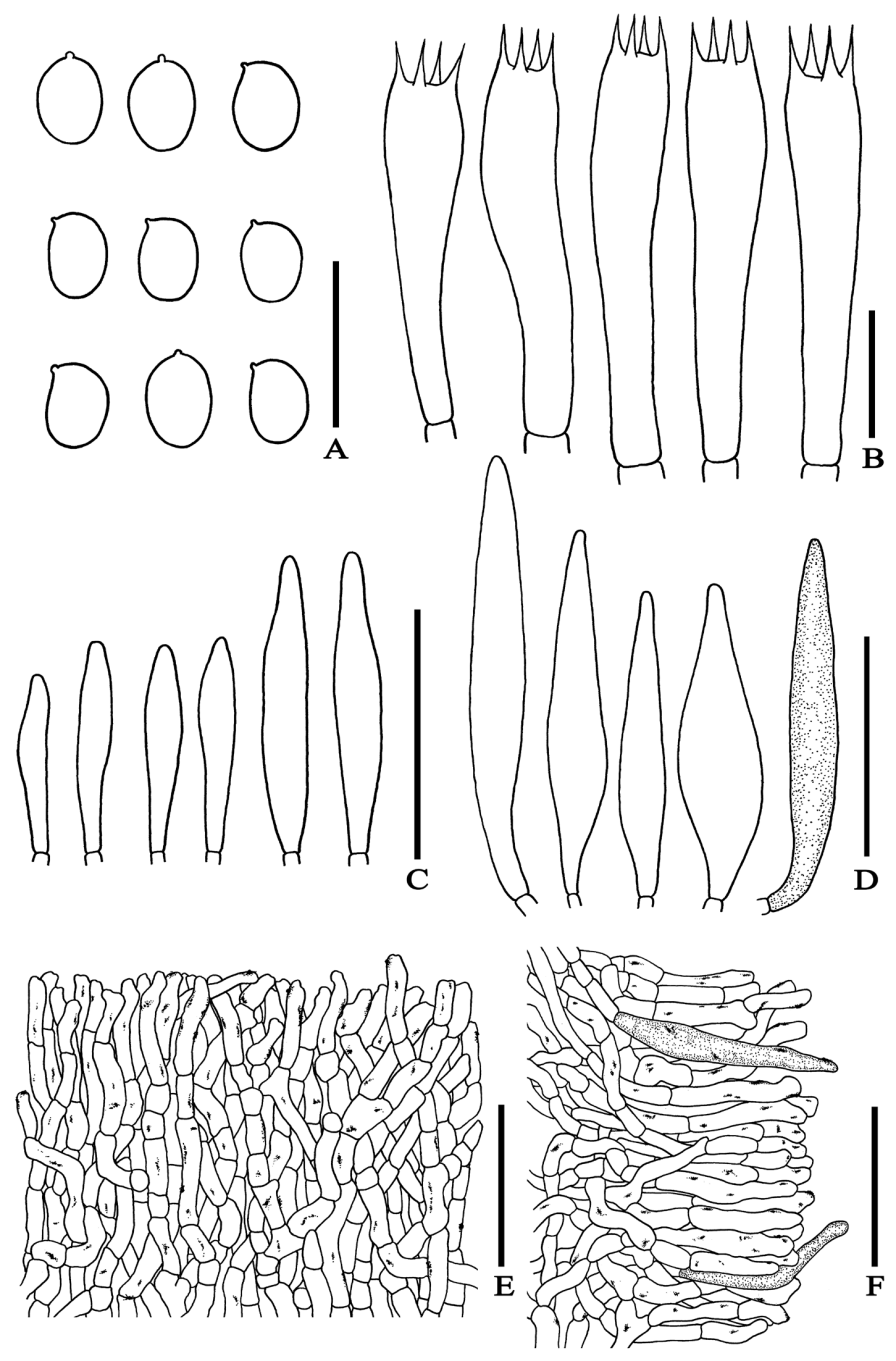
Microscopic features of *Erythrophylloporussuthepensis***A** basidiospores **B** basidia **C** cheilocystidia **D** pleurocystidia **E** pileipellis **F** stipitipellis showing some dark caulocystidia mixed with slightly rough, cylindrical to irregular hyphae. – Scale bars: 10 µm (**A–B**); 50 µm (**C–F**). All drawings were made from the type (SV0236).

Figs 2B , 3B , 4B and 6

##### Holotype.

THAILAND, Chiang Mai Province, Muang District, Doi Suthep-Pui National Park, 18°48'47"N, 98°55'56"E, elev. 645 m, 25 August 2015, *S. Vadthanarat*, SV0236, (holotype CMUB, isotype BKF, BR).

##### Etymology.

Refers to the type locality Doi Suthep.

##### Description.

*Basidiomata* stipitate-pileate with lamellate hymenophore, small-sized. *Pileus* (1.0–)2.5– 3.5 cm in diameter, subumbonate with involute margin at first, becoming convex to plano-convex with inflexed margin; surface even with some small pustules, tomentose, dull, slightly moist, yellow (3–4A4– 5) becoming light orange to orange-red (5–6A5–7 to 7–8A–B7–8) with patches of light yellow to light orange (4–5A5–6) becoming brownish-orange to dull red (7B–C8 to 8B–D8) with age, the colour of the margin when young clearly paler than the rest of the pileus, bluing when bruised. *Pileus context* 2–3 mm thick half-way to the margin, tough, yellowish-orange (4A5), unchanging when bruised. *Stipe* 2.5– 4.5 × 0.3– 0.8 cm, central, slightly curved, terete, dull, dry, yellowish-orange (2A6–7) with greyish-orange (5–6 B 7–8) coarse scales at first, then light yellow or reddish-yellow to brownish-orange (4A/B5–6 to 7C6) with brownish-red to reddish-dark brown (7F4–5, 8C7–8, 8F5–7) scales, sub-bulbous, with bright yellow to greyish-yellow (2A6–7 to 3A/B5–6) sparse basal mycelium that extends half-way up the stipe. *Stipe context* solid, tough, reddish-yellow (4A6) near the pileus then paler to light yellow (4A5) near the base, unchanging when bruised. *Hymenophore* lamellate; lamellae decurrent, subdistant, slightly thick, with sinuate edge, of varying lengths, 26–34 lamellae, with 4–6 different lengths of lamellullae, 4–5 mm wide half-way to margin, brownish-orange (7C7–8) with deep yellow to orange (4–5A7–8) edge, bluish-grey when looking tangentially to the surface, bluing when bruised. *Odour* rubbery. *Taste* mild with rubbery texture. *Spore print* olivaceous brown (4F5).

*Macrochemical reactions*. KOH orange-brown on pileus and stipe surface; yellowish-brown on pileus and stipe context and hymenophore. NH_4_OH yellowish-brown on pileus and stipe surface and hymenophore; yellowish on pileus and stipe context.

*Basidiospores* [218/4/2] (4.6–)4.8–5.2–5.7(–5.9) × (3.5–)3.6–4–4.3(–4.5) µm, *Q* = (1.15–)1.21–1.32–1.44(–1.57); from the type (SV0236) (4.6–)4.8–5.2–5.7(–5.9) × (3.5–)3.6–3.9–4.4(–4.5) µm, *Q* = (1.15–)1.21–1.32–1.43(–1.57), *N* = 80, broadly ellipsoid to subglobose, smooth under light microscope and SEM, yellowish to pale brown in water, hyaline in 5% KOH, thin-walled, inamyloid. *Basidia* 4-spored, (24.7–)25.3–31.1–35.8(–35.9) × (5.3–)5.3–6.6–7.5(–7.5) µm, narrowly clavate to subcylindrical, attenuated towards the base, clampless, hyaline to yellowish hyaline in water, Melzer’s reagent and 5% KOH; *sterigmata* up to 4.5 µm long. *Cheilocystidia* (37.3–)37.9–51–63.8(–64.1) × (5.3–)5.4–8.5–12.4(–13.7) µm, narrowly conical to narrowly fusiform with obtuse apex, projecting up to 25 µm, thin-walled, smooth, yellowish-hyaline in water, hyaline in 5% KOH and NH_4_OH, inamyloid, more or less forming a sterile edge . *Pleurocystidia* (46.5–)49.2–68.9–95.2(–99.3) × (9.3–)9.6–12.6–18.9(–20) µm, abundant, narrowly conical with obtuse apex, projecting up to 28 µm, thin-walled, mostly yellowish hyaline in water and hyaline in 5% KOH and NH_4_OH, some containing yellowish-brown to dark brown pigments in water and yellowish-pale brown in 5% KOH and NH_4_OH, inamyloid, arising more or less deeply in the subhymenium or from hymenophoral trama. *Hymenophoral trama* subregular near the pileus context becoming slightly divergent near the edge, 46–192 µm wide, widest near the pileus context then getting narrower when close to the edge, composed of clampless hyphae 2.5–7.5 µm wide, pinkish-red hyaline in water, especially at the centre of the trama, yellowish hyaline to hyaline in 5% KOH and NH_4_OH. *Pileipellis* a palisadoderm to trichoderm 71–119 µm thick, composed of slightly thick-walled, cylindrical to irregular hyphae with fine encrustation on the wall, terminal cells 12–46 × 3.5–9 µm with pointed to notched apex or sometimes truncated apex, with 6–15(–28) µm short cells at the base, hyaline or yellowish-orange hyaline to orange hyaline hyphae with scattered fine encrustation on the wall when observed in water, hyaline to yellowish hyaline in 5% KOH and NH_4_OH, inamyloid. *Pileus context* composed of slightly thick-walled, strongly interwoven hyphae, 5–8.5 µm wide, inamyloid. *Stipitipellis* a disrupted palisadoderm perpendicular to the stipe axis, 47–123 µm thick, composed of slightly thick-walled, cylindrical to irregular hyphae with fine encrustations on the wall, yellow to yellowish-orange, intermixed with mostly yellowish hyaline to yellowish-brown hyphae in 5% KOH and NH_4_OH, terminal cells 14–47 × 4–8.5 µm with variously notched apex. *Caulocystidia* mixed in a group with the stipitipellis hyphae, same shape and size as the pleurocystidia, dark brown in water, paler in 5% KOH and NH_4_OH. *Stipe context* composed of parallel, densely packed, 4–9.5 µm wide hyphae, hyphae wall with scattered fine encrustations when observed in water. *Clamp connections* not seen in any tissue.

##### Habit and habitat.

On soil, gregarious (up to 10 basidiomata) in dipterocarp forest dominated by *Dipterocarpustuberculatus*, *D.obtusifolius*, *Shoreaobtusa* and *S.siamensis*, mixed with scattered fagaceous trees.

##### Known distribution.

Currently known only from Doi Suthep-Pui National Park, Chiang Mai Province, northern Thailand.

##### Additional specimens examined.

– THAILAND, Chiang Mai Province, Meuang District, Doi Suthep-Pui National Park, 18°48'05"N, 98°55'40"E, elev. 800 m, 17 May 2015, *O. Raspé*, OR0615B (CMUB, BKF, BR).

##### Remarks.

*Erythrophylloporussuthepensis* is characterised by the following combination of features: yellow to light orange to orange red to brownish-orange to dull red pileus; brownish-orange lamellae with deep yellow to orange edge; the colour of the lamellae appears more bluish-grey when observed from an oblique angle to the surface; pileus surface and lamellae turning blue when bruised; some pleurocystidia containing yellowish-brown to dark brown pigments in water; basidiospores that are smaller or shorter (4.6–5.9 × 3.5–4.5 µm) than the other *Erythrophylloporus* species (*E.aurantiacus* = 6.0–7.5 × 4–5.5µm; *E.cinnabarinus* = 5.5–7 × 4.5–5.5 µm; *E.fagicola* = 6.5–11 × 4–7.5 µm; *E.paucicarpus* = 5.9–8 × 4.1–6 µm) (Halling et al. 1999, Montoya and Bandala 2011, Zhang and Li 2018).

Morphologically, *E.suthepensis* is quite similar to *E.cinnabarinus* in that they have similar colours in pileus and lamellae; the lamellae in both species also turn more or less blue to dark blue when bruised. *Erythrophylloporussuthepensis* and *E.cinnabarinus* are also similar, based on some pleurocystidia containing yellowish-brown to dark brown pigments, but those features are not found in *E.paucicarpus* and in the two New World *Erythrophylloporus* species (Halling et al. 1999, Montoya and Bandala 2011). However, the pleurocystidia containing brown pigments seem to be more frequent in *E.cinnabarinus*, which also has, on average, larger basidiospores than *E.suthepensis* (Zhang and Li 2018).

The pinkish-red hymenophoral trama of *E.suthepensis* was not found in either *E.paucicarpus* or in the two American *Erythrophylloporus* species. In our observation of the two American specimens (*E.aurantiacus* voucher REH7271 and *E.fagicola* voucher Garay215), we found that the hymenophoral trama was yellowish hyaline when observed in water. The original description of *E.cinnabarinus* does not mention the colour of the hymenophoral trama and we could not obtain a specimen to observe this character. However, other morphological characters and phylogenetic evidence are enough to differentiate *E.suthepensis* from *E.cinnabarinus*.

Our phylogenetic analyses of a four-gene dataset revealed that *Phylloporusaurantiacus* from Costa Rica and *P.fagicola* from Mexico clustered in the *Erythrophylloporus* clade with high support (BS = 100% and PP = 1). Both species possess the distinctive morphological characters of *Erythrophylloporus*, which include yellowish-orange to reddish-orange basidiomata, orange to orange brown lamellae, bright yellow basal mycelium, ovoid or ellipsoid to broadly ellipsoid basidiospores with smooth surface and subcylindrical to subfusoid to ventricose cheilocystidia and pleurocystidia (Halling et al. 1999, Montoya and Bandala 2011). Therefore, the following two new combinations are proposed:

#### Erythrophylloporus aurantiacus

Taxon classificationFungiBoletalesBoletaceae

(Halling & G.M. Muell.) Raspé & Vadthanarat comb. nov.

823607

##### Basionym.

*Phylloporusaurantiacus* Halling & G.M. Mueller, Mycotaxon 73: 64 (1999)

##### Specimen examined.

– COSTA RICA. Near town of Palo Verde, elev. 1600 m, 11 June 1994, Halling 7271 (NY).

#### Erythrophylloporus fagicola

Taxon classificationFungiBoletalesBoletaceae

(Montoya & Bandala) Raspé & Vadthanarat comb. nov.

823608

##### Basionym.

*Phylloporusfagicola* Montoya & Bandala, Mycotaxon 117: 10 (2011)

##### Specimen examined.

– MEXICO. Veracruz: Mpio. Acatlán, Acatlán Volcano, 29 September 2009, Garay 215 (XAL).

### Key to the species in *Erythrophylloporus*

**Table:** 

1	Growing in North or Central America	**2**
–	Growing in Southeast Asia or in tropical to subtropical China	**3**
2	Bluing of the context when exposed; basidiospores ellipsoid to oblong, obtuse, 6.5–11 × 4–7.5 µm; pleurocystidia somewhat thick-walled (0.8–3.5 µm thick)	*** E. fagicola ***
–	Context unchanging when exposed; basidiospores ovoid to subellipsoid, 6.0–7.5 × 4–5.5 µm; pleurocystidia thin-walled	*** E. aurantiacus ***
3	Yellowish-orange lamellae slightly reddening when bruised; context slowly or slightly reddening when exposed	*** E. paucicarpus ***
–	Brownish-orange or orange, deep orange, reddish-orange to orange red lamellae bluing to greyish-green when bruised; context unchanging to gradually turning dark violet, blackish to dark blue	**4**
4	Basidiospores 4.6–5.9 × 3.5–4.5 µm, broadly ellipsoid to subglobose; cystidia mostly hyaline, only some containing yellowish-brown to dark brown pigments.	*** E. suthepensis ***
–	Basidiospores 5.5–7 × 4.5–5.5 µm, broadly ellipsoid, ellipsoid to nearly ovoid; cystidia usually containing yellowish-brown pigments	*** E. cinnabarinus ***

## Discussion

Both phylogeny and morphology support the placement of the two new species from Thailand, *E.paucicarpus* and *E.suthepensis* in the genus *Erythrophylloporus*. Phylogenetically, both species were highly supported in the *Erythrophylloporus* clade close to *E.cinnabarinus* (typus generis). Morphologically, they are characterised by having yellowish-orange to reddish- to brownish-orange basidiomata with bright yellow basal mycelium and smooth, ellipsoid, broadly ellipsoid to subglobose basidiospores. The other lamellate Boletaceae in *Phylloporus*, *Phylloboletellus* and *Phylloporopsis* are solely similar to the new species by having a lamellate hymenophore instead of a poroid hymenophore. However, *Phylloporus* differs from *Erythrophylloporus* species by having whitish- to yellowish-pale brown basidiomata with yellow to golden-yellow lamellae, with off-white to whitish to yellow basal mycelium and most species in the genus have basidiospores with more or less bacillate ornamentation under SEM (Neves & Halling 2010, Neves et al. 2012, Zeng et al. 2013). The single *Phylloboletellus* species, *Ph.chloephorus* Singer differs from *Erythrophylloporus* by having longitudinally ridged basidiospores (Bandala et al. 2004). The sole species of *Phylloporopsis*, *Phy.boletinoides*, differs by having beige to olive-cream or olive buff lamellate to subporoid hymenophore, with anastomosing and interveined gills and basal mycelium whitish to yellowish (Farid et al. 2018). Moreover, those genera are phylogenetically distant from *Erythrophylloporus*. (Bandala et al. 2004, Neves & Halling 2010, Neves et al. 2012, Zeng et al. 2013, Farid et al. 2018).

Interestingly, *Phylloporuscoccineus* Corner, described from Singapore (Corner 1970), is similar to *Erythrophylloporus* species, in that it produces crimson to scarlet, lamellate basidiomata with orange to orange-red lamellae and yellow basal mycelium, broadly ellipsoid to subglobose and smooth basidiospores. It probably should also be transferred to *Erythrophylloporus*, but we refrain from doing so until specimens become available for molecular study. According to the protologue of *P.coccineus*, it differs from the newly described Asian species of *Erythrophylloporus* by having larger basidiospores (7.5–10 × 6.5–8 µm), larger cheilocystidia (70–120 × 10–18 µm) and larger caulocystidia (up to 200 × 10–16 µm) (Corner 1970).

*Erythrophylloporus* species formed two clades, an Asian species clade (BS = 65% and PP = 1) and a New World species clade (BS = 100% and PP = 1) (Fig. 1). The Asian one contains three species, *E.cinnabarinus*, *E.paucicarpus* and *E.suthepensis*, while the American clade contains the remaining two species *E.aurantiacus* and *E.fagicola*. *Erythrophylloporusaurantiacus* and *E.fagicola* seem to be genetically very close to each other, much closer than the species in the Asian clade. Only morphological differences between the two species were used to separate them from each other. *Erythrophylloporusfagicola* produces larger basidiospores than *E.aurantiacus* and pleurocystidia are somewhat thick-walled (0.8–3.5 µm thick) in *E.fagicola*, whereas they are thin-walled in *E.aurantiacus* and the latter has non-staining context, whereas the former has a cyanescent context. However, the descriptions were based on a limited number of collections and more samples are desirable to verify whether the morphological traits observed are good characters differentiating the two species or merely extremes of a continuum in morphological variation within a single species.

Regarding the phylogenetic affinities of *Erythrophylloporus*, Zhang and Li (2018) reported that it was likely close to the genus *Rugiboletus* G. Wu & Zhu L. Yang and *Lanmaoa* G. Wu & Zhu L. Yang, based on a multilocus dataset of nrLSU, *tef*1, *rpb*1 and *rpb*2, although this relationship was not supported in their phylogram. In our phylogeny, based on a multilocus dataset of *atp*6, *tef*1, *rpb*2 and *cox*3, with wider taxon sampling, *Erythrophylloporus* also clustered within the *Pulveroboletus* group, but was sister to *Singerocomus* with high bootstrap support (96%) but relatively weak posterior probability support (0.86). *Singerocomus* contains three species, *S.atlanticus* A.C. Magnago, *S.inundabilis* (Singer) T.W. Henkel and *S.rubriflavus* T.W. Henkel & Husbands that have some similar morphological characters to *Erythrophylloporus*, including red-orange to red pileus and light yellow basal mycelium. The three existing *Singerocomus* species are clearly different from all known *Erythrophylloporus* species by having a poroid, non-cyanescent hymenophore (Henkel et. al. 2016, Magnago et al. 2018). However, the hymenophore structure (lamellate vs. poroid) is not sufficient to separate genera in Boletaceae. *Phylloporus* currently contains both lamellate and poroid species, although some poroid species have already been transferred to another genus, *Hourangia* (Zhu et al. 2015). Phylogenetic analyses, including the remaining poroid *Phylloporus* species, are needed to verify their taxonomic position.

*Erythrophylloporus* putatively forms ectomycorrhizal associations with trees in family Fagaceae, including the genera *Fagus*, *Lithocarpus* and *Quercus* (Neves and Halling 2010, Montoya and Bandala 2011, Zhang and Li 2018). The two Thai *Erythrophylloporus* species were found in forests dominated by Dipterocarpaceae trees, mainly *Dipterocarpus*, including *D.tuberculatus*, *D.obtusifolius* and *Shorea*, including *S.obtusa* and *S.siamensis*. However, some *Quercus* and *Lithocarpus* trees (Fagaceae) were also observed in the vicinity and could also be the ectomycorrhizal partners. Further study is needed to confirm the ectomycorrhizal relationships of *Erythrophylloporus*.

## Supplementary Material

XML Treatment for Erythrophylloporus

XML Treatment for Erythrophylloporus paucicarpus

XML Treatment for Erythrophylloporus suthepensis

XML Treatment for Erythrophylloporus aurantiacus

XML Treatment for Erythrophylloporus fagicola
